# Supplementary figures and images for: Yeast filamentation signaling is connected to a specific substrate translocation mechanism of the Mep2 transceptor
Source: PLoS Genet. 2020 Feb 18;16(2):e1008634. doi: 10.1371/journal.pgen.1008634 (PMC7048316; doi:10.1371/journal.pgen.1008634)

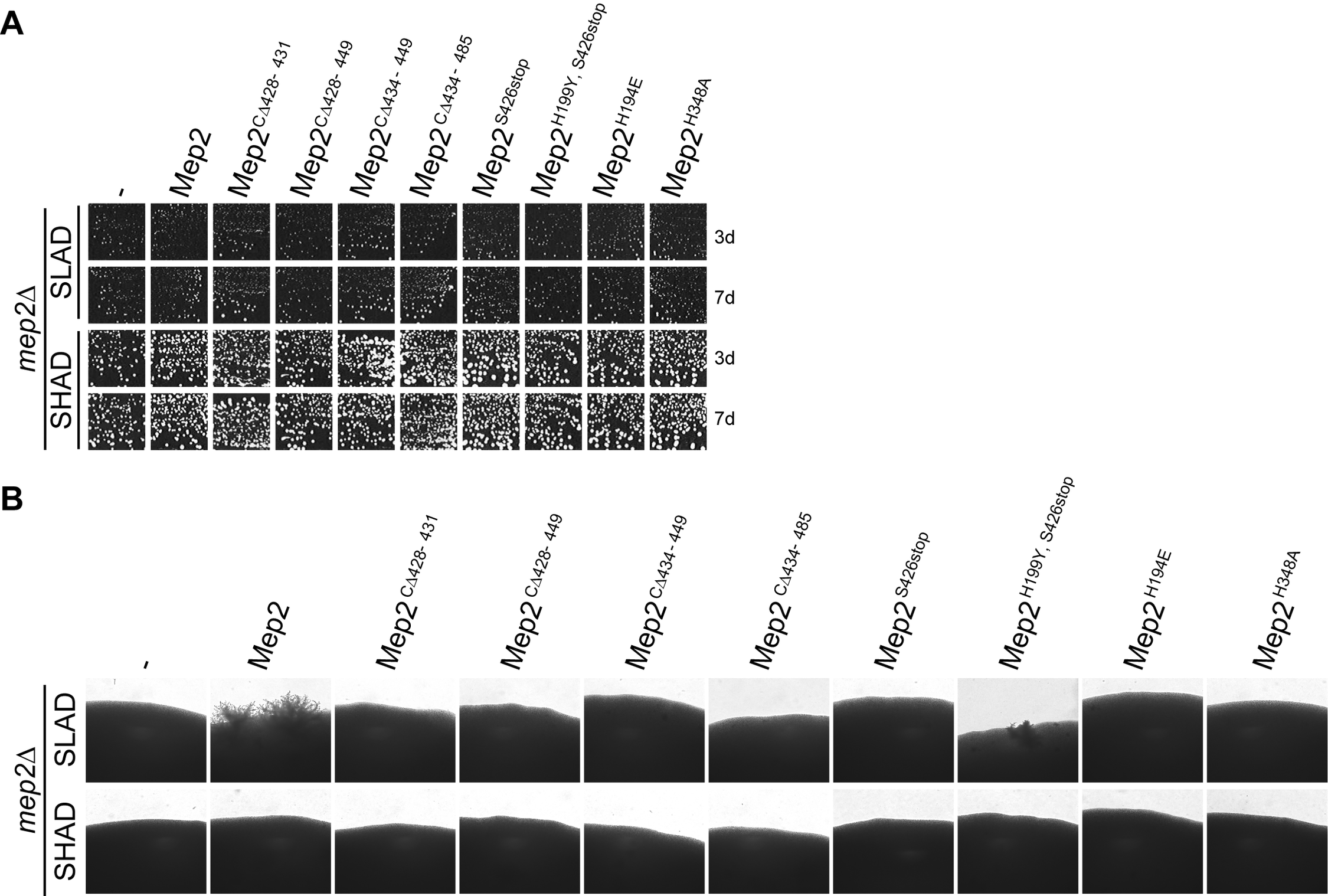

Supplement: S1 Fig — (A-B) Homozygous diploid mep2Δ (ZAB2) cells were transformed with the pFL38 empty plasmid (-) or with YCpMep2, YCpMep2S426stop, YCpMep2CΔ428–431, YCpMep2CΔ428–449, YCpMep2CΔ434–449, YCpMep2CΔ434–485, YCpMep2H199Y, S426stop, YCpMep2H194E and YCpMep2H348A. (A) Growth tests on SLAD and SHAD media at day 3 (3d) and 7 (7d) at 29°C. (B) Pseudohyphal growth tests on SLAD and SHAD media at day 7 at 29°C. (TIF) [file pgen.1008634.s001.tif]
