# Supplementary material for: Yeast filamentation signaling is connected to a specific substrate translocation mechanism of the Mep2 transceptor
Source: PLoS Genet. 2020 Feb 18;16(2):e1008634. doi: 10.1371/journal.pgen.1008634 (PMC7048316; doi:10.1371/journal.pgen.1008634)
Supplement: S2 Fig — Two datasets were retrieved from the UniRef100 database by Blast searches with the ScMep1 (sp:P40260) and ScMep2 (sp:P41948) as searching sequences, respectively. No sequence was in common to the two datasets by considering the 350 top hits reported. Multiple sequence alignments were performed with the ClustalW2 program. Sequences containing too many gaps in the multiple alignments were removed, resulting in 348 sequences for both pools of sequences. Sequences were subdivided according secondary structures and subdomain limits, giving the following structural segments, from N- to C-terminal, the external N-terminus, the transmembrane helix #1 (TM1), the cytoplasmic loop #1 (CL1), TM2, the external loop #2 (EL2), TM3, CL3, TM4, EL4, TM5, CL5, TM6, EL6, TM7, CL7, TM8, EL8, TM9, CL9, TM10, EL10, TM11, the cytoplasmic C-terminal proximal linker, the cytoplasmic enhancer subdomain, the cytoplasmic C-terminal linker subdomain, the cytoplasmic autoinhibitory subdomain and the distal part of the cytoplasmic C-terminus. Note that the CTD of ScMep1 was subdivided according sequence comparison with ScMep2. The WebLogo server (https://weblogo.berkeley.edu) was then used to generate the different plots, one for each structural segment. The logo consists of amino acid stack for each position in the aligned sequence dataset. The height of the stack indicates the sequence conservation at that position (maximum = 4 bits), while the height of the amino acid one-letter symbol within the stack shows the relative frequency of the amino acid at that position. The color scheme is aromatic amino acids (F, H, W, Y) in green, polar (N, Q, S, T) in purple, negatively charged (D, E) in red, positively charged (K, R) in blue, aliphatic (A, I, L, M, V) in black, cysteine (C) in brown, proline (P) in grey and glycine (G) in orange. Sequence numbering is according ScMep1 or ScMep2 sequences. (PDF) [file pgen.1008634.s002.pdf]

External N-terminus

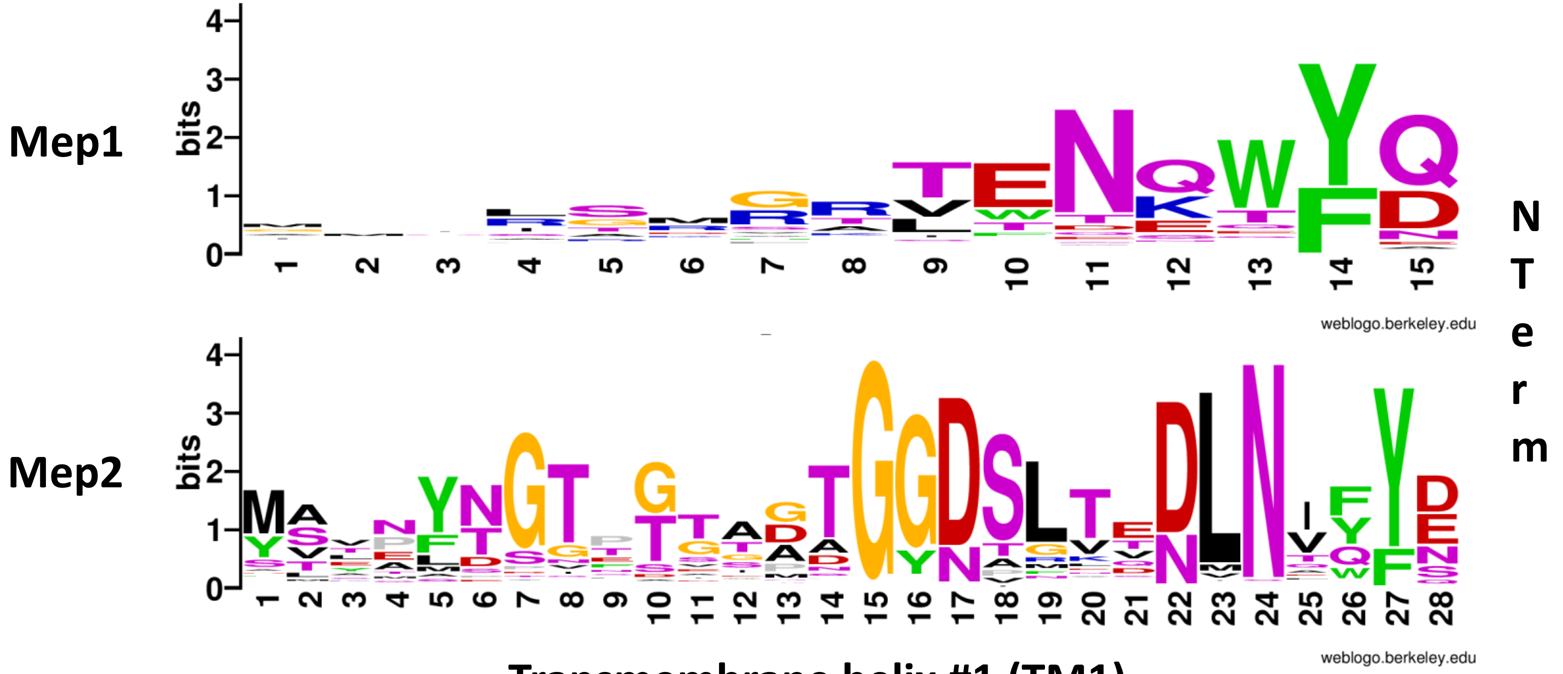

Transmembrane helix #1 (TM1)

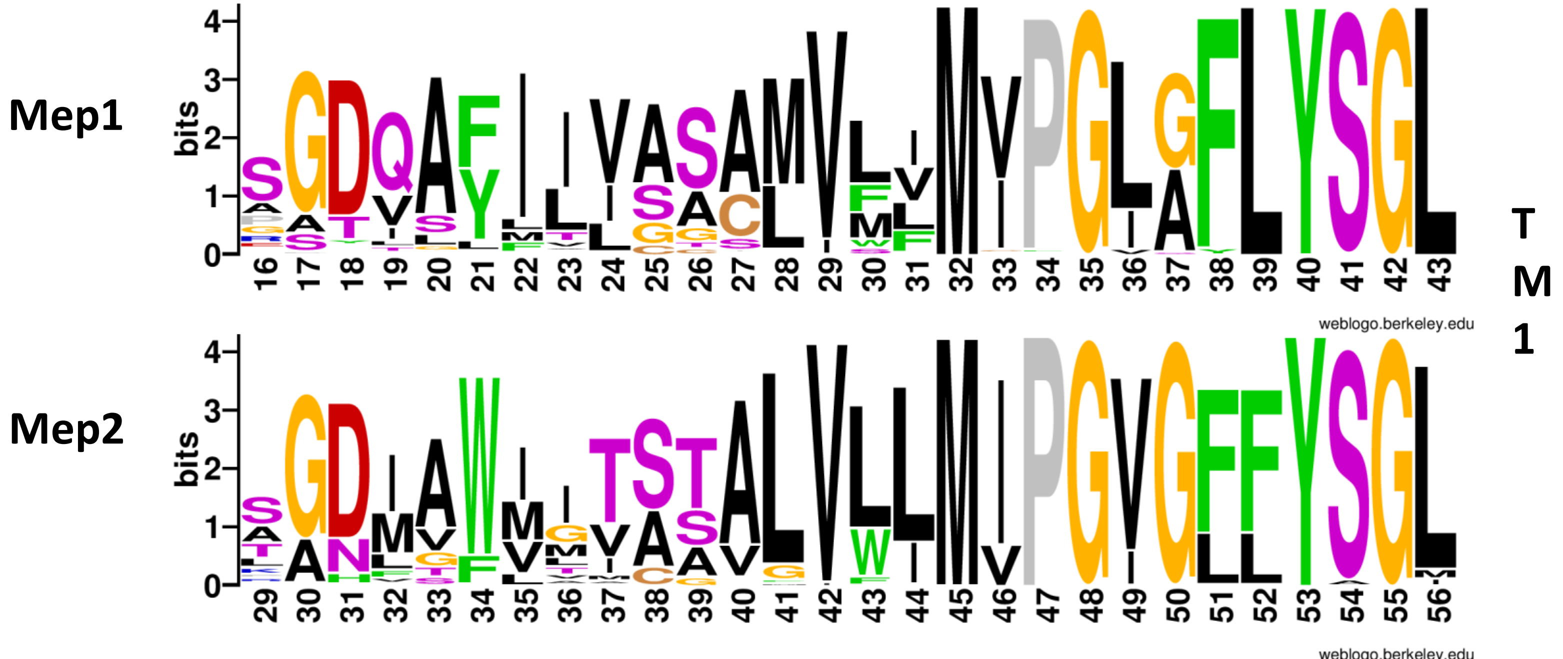

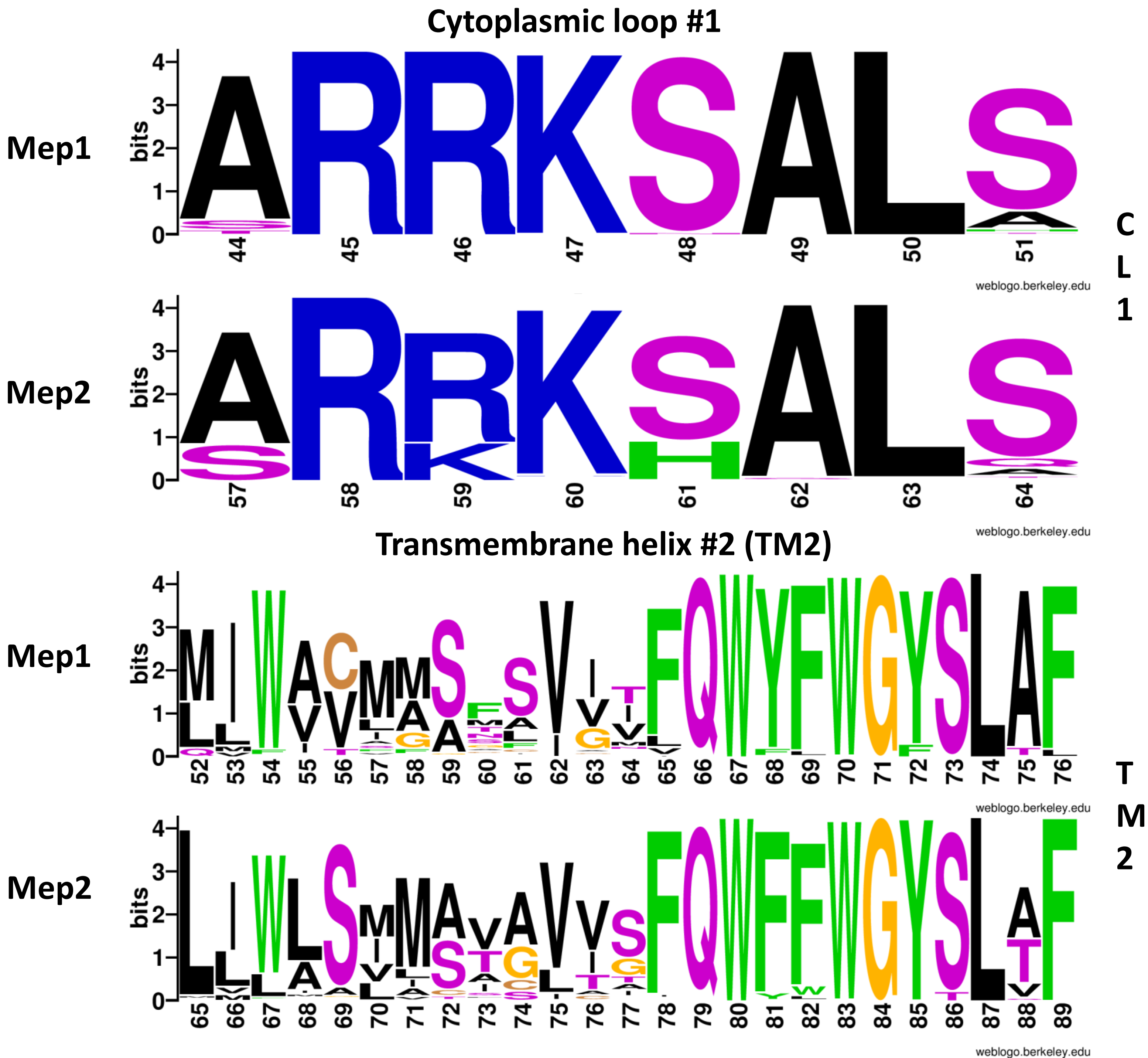
Cytoplasmic loop #1
Mep1
Mep2
CL1
Transmembrane helix #2 (TM2)
Mep1
Mep2
TM2
bits
weblogo.berkeley.edu

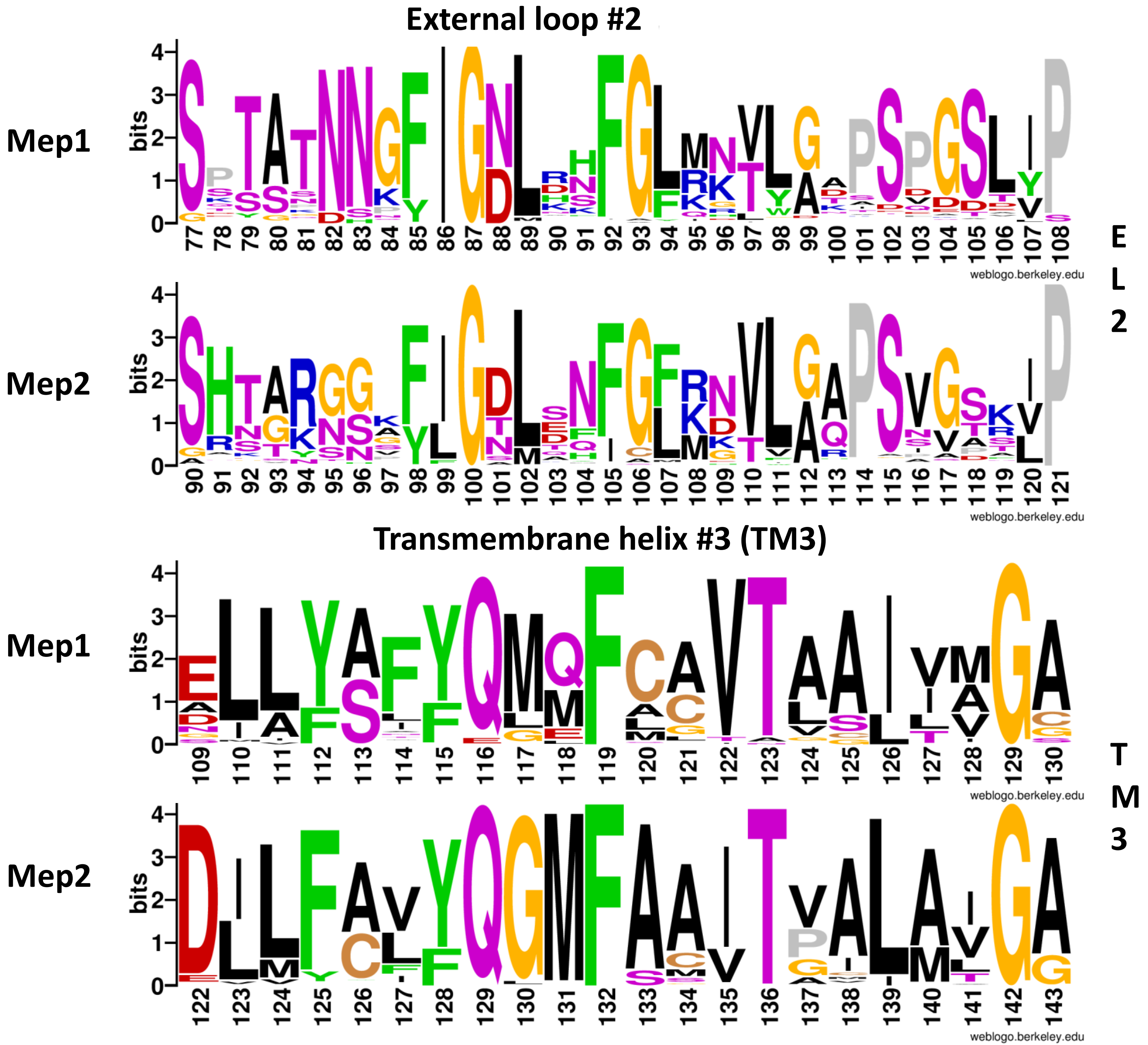
External loop #2
Mep1
Mep2
EL2
Transmembrane helix #3 (TM3)
Mep1
Mep2
TM3
bits
weblogo.berkeley.edu

Cytoplasmic loop #3

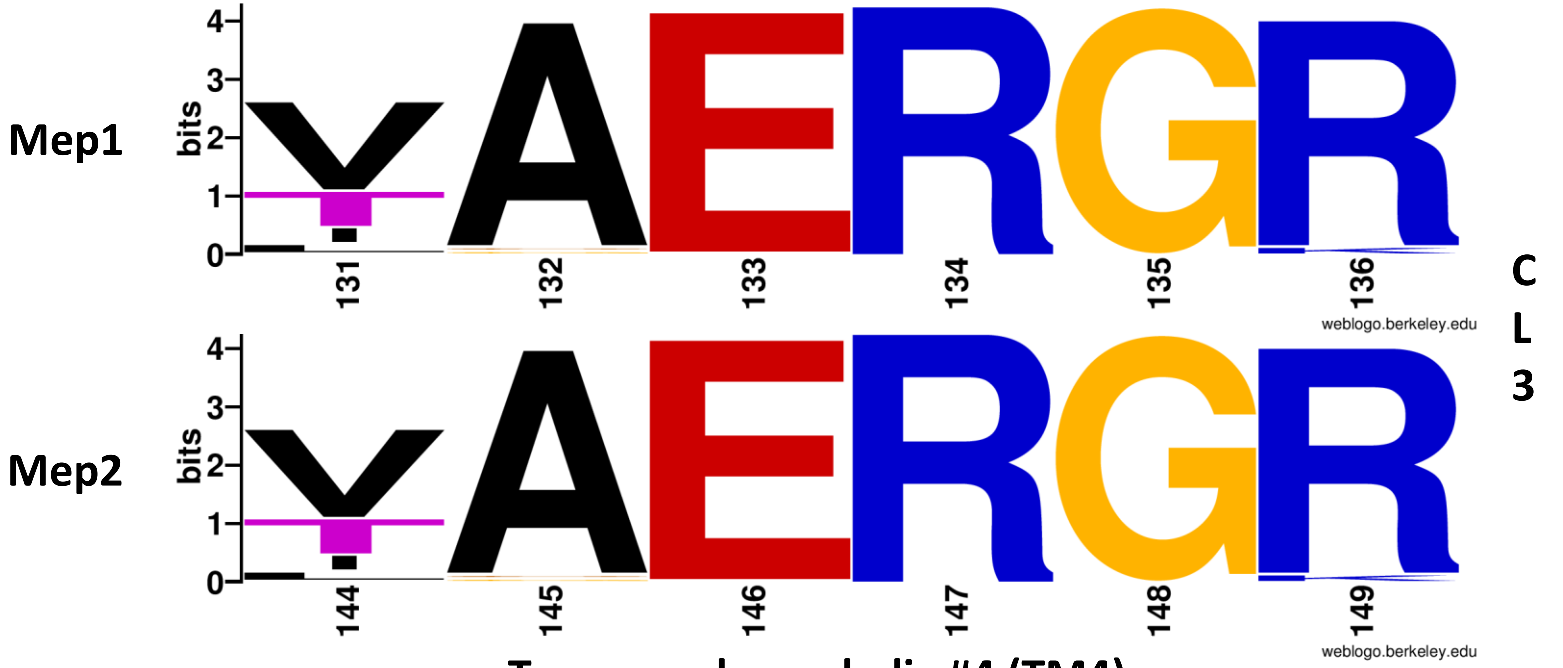

Transmembrane helix #4 (TM4)

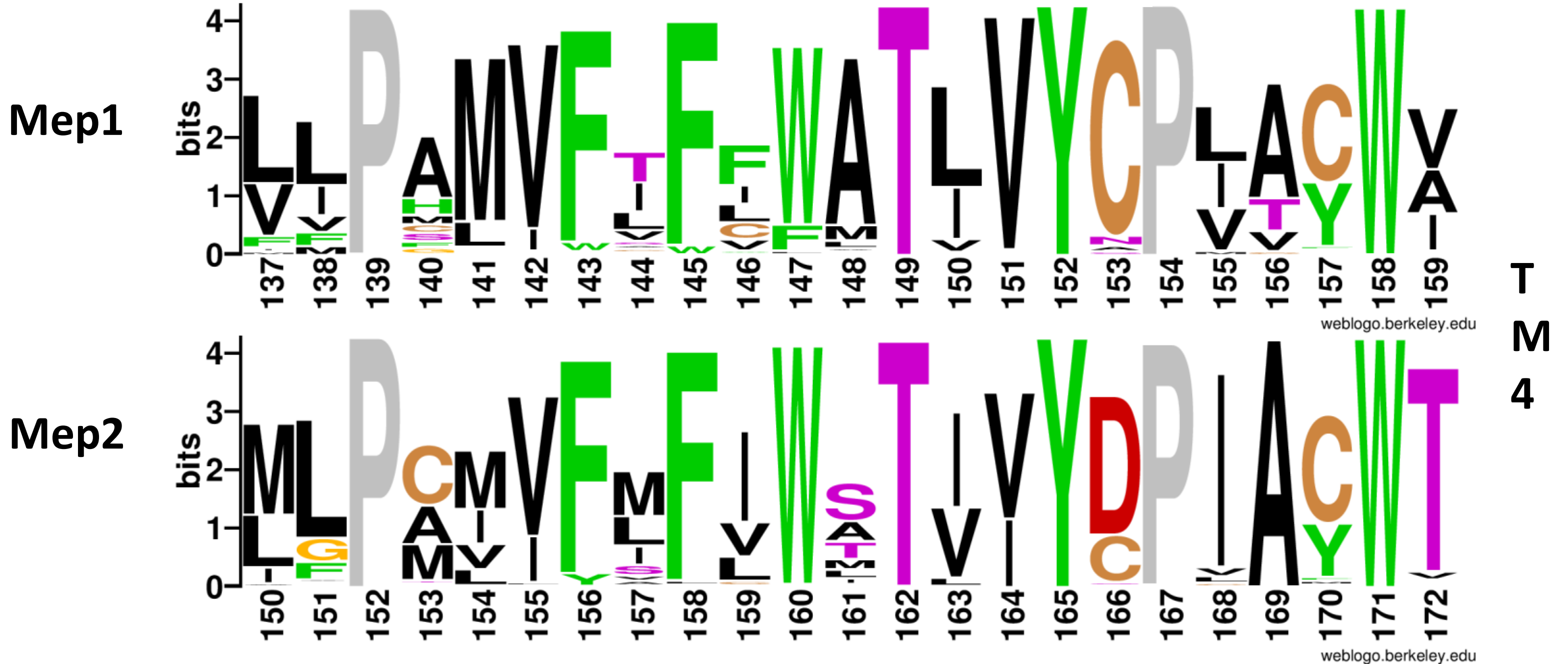

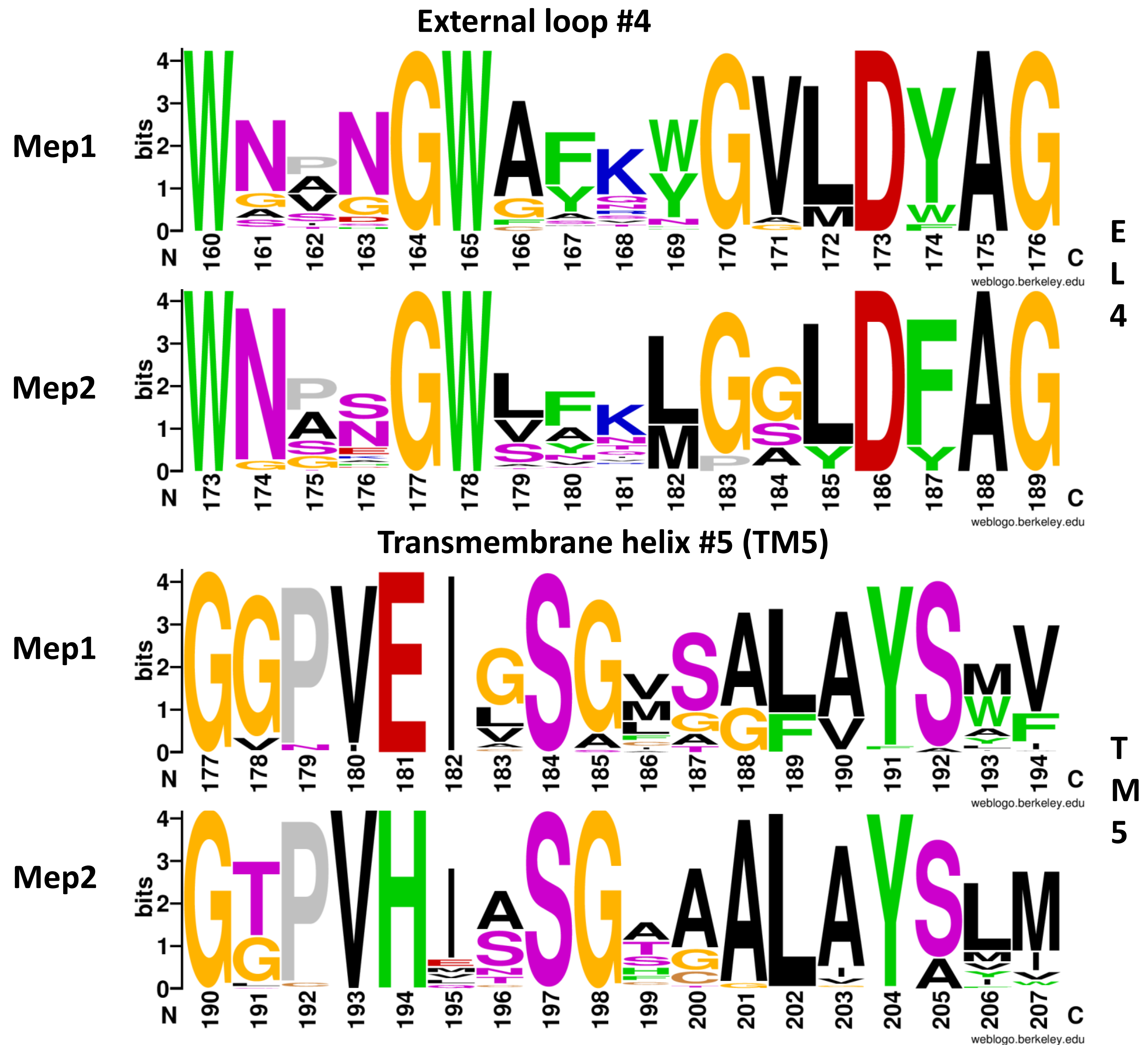
External loop #4
Mep1
Mep2
bits
weblogo.berkeley.edu
E
L
4
Transmembrane helix #5 (TM5)
T
M
5

Cytoplasmic loop #5

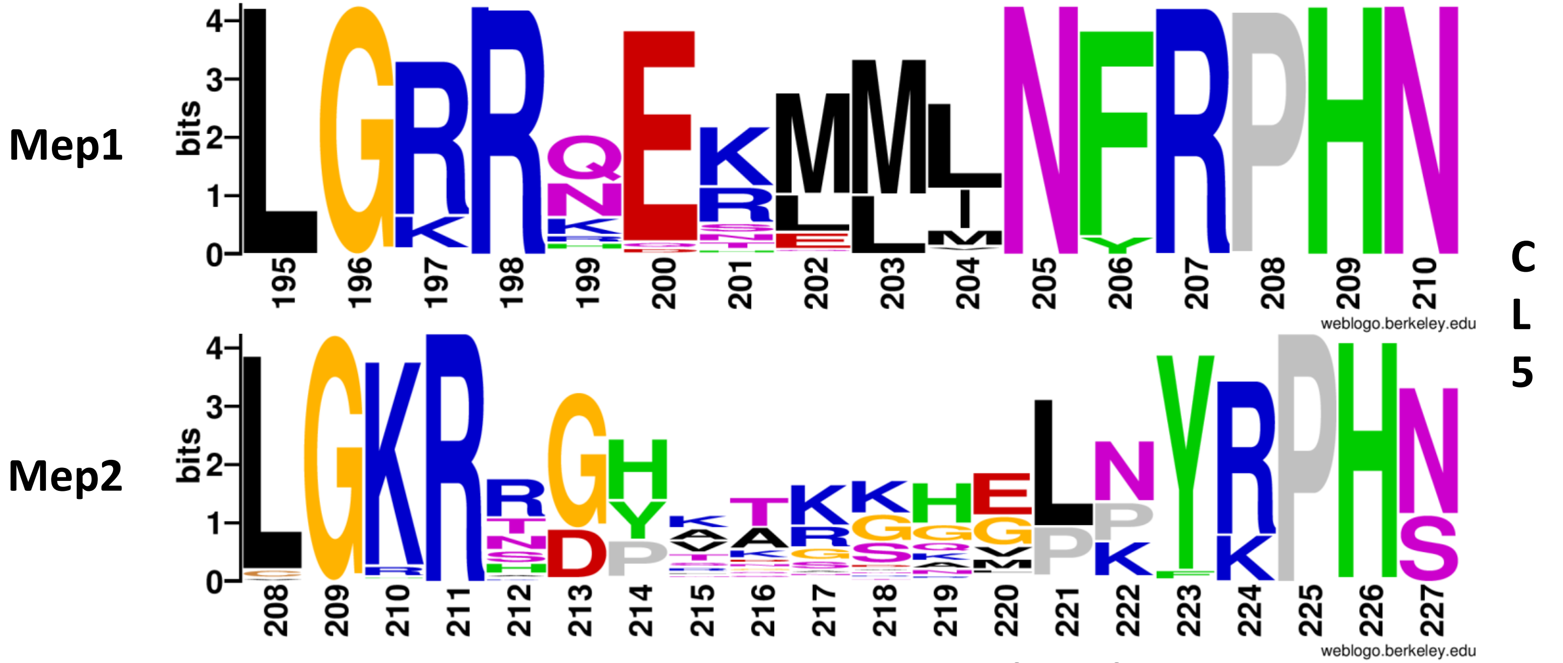

Transmembrane helix #6 (TM6)

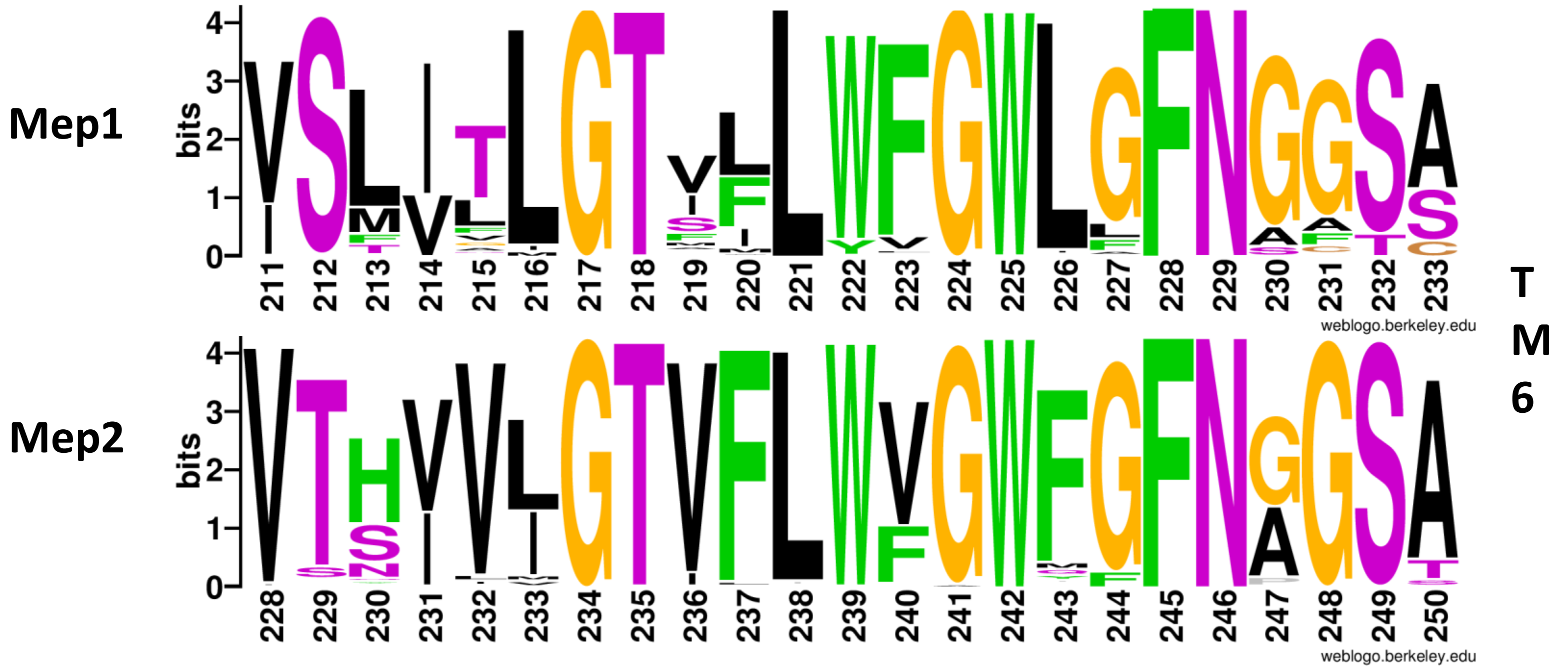

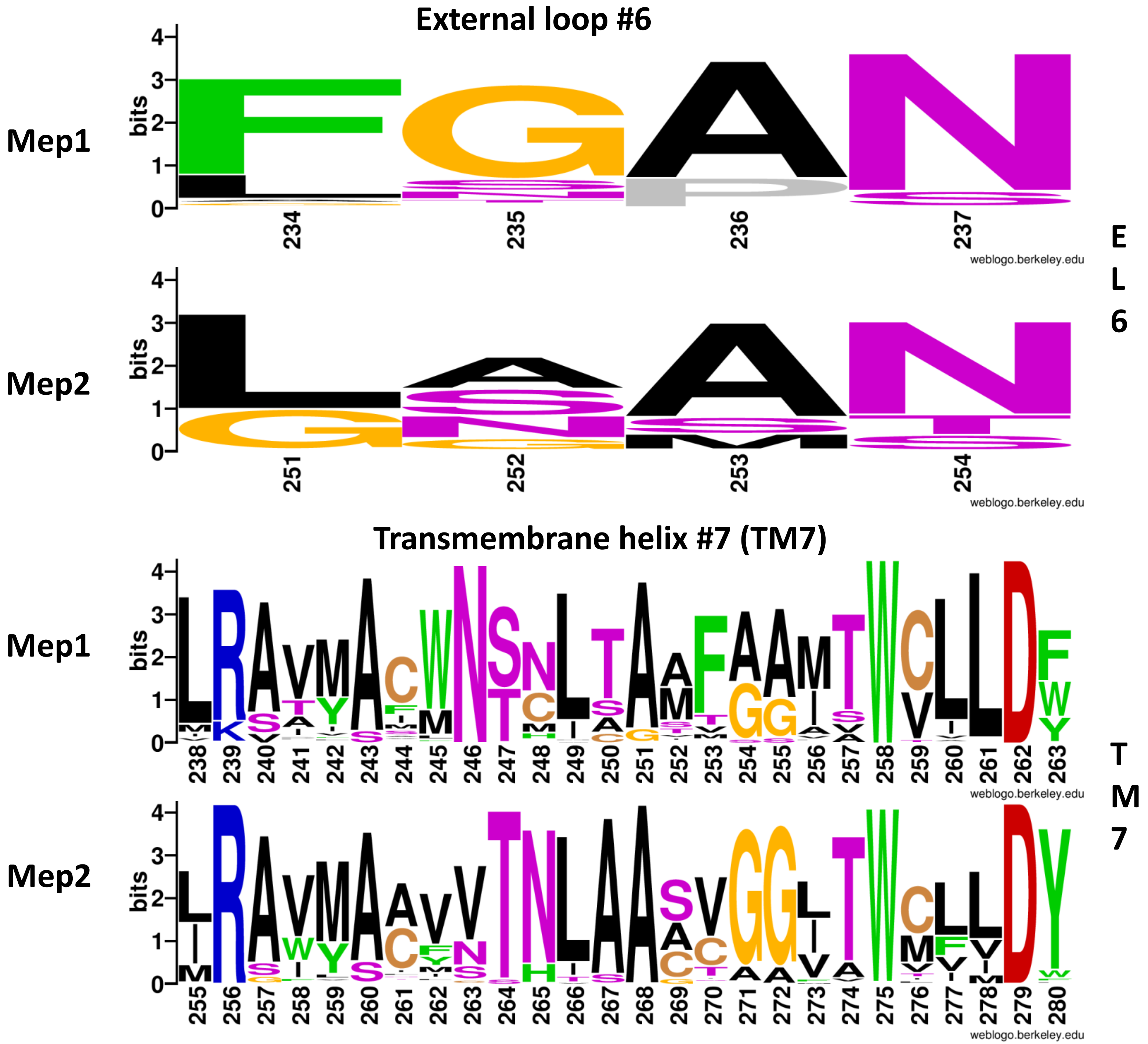
External loop #6
Mep1
Mep2
E
L
6
Transmembrane helix #7 (TM7)
Mep1
Mep2
T
M
7
bits
weblogo.berkeley.edu

Cytoplasmic loop #7

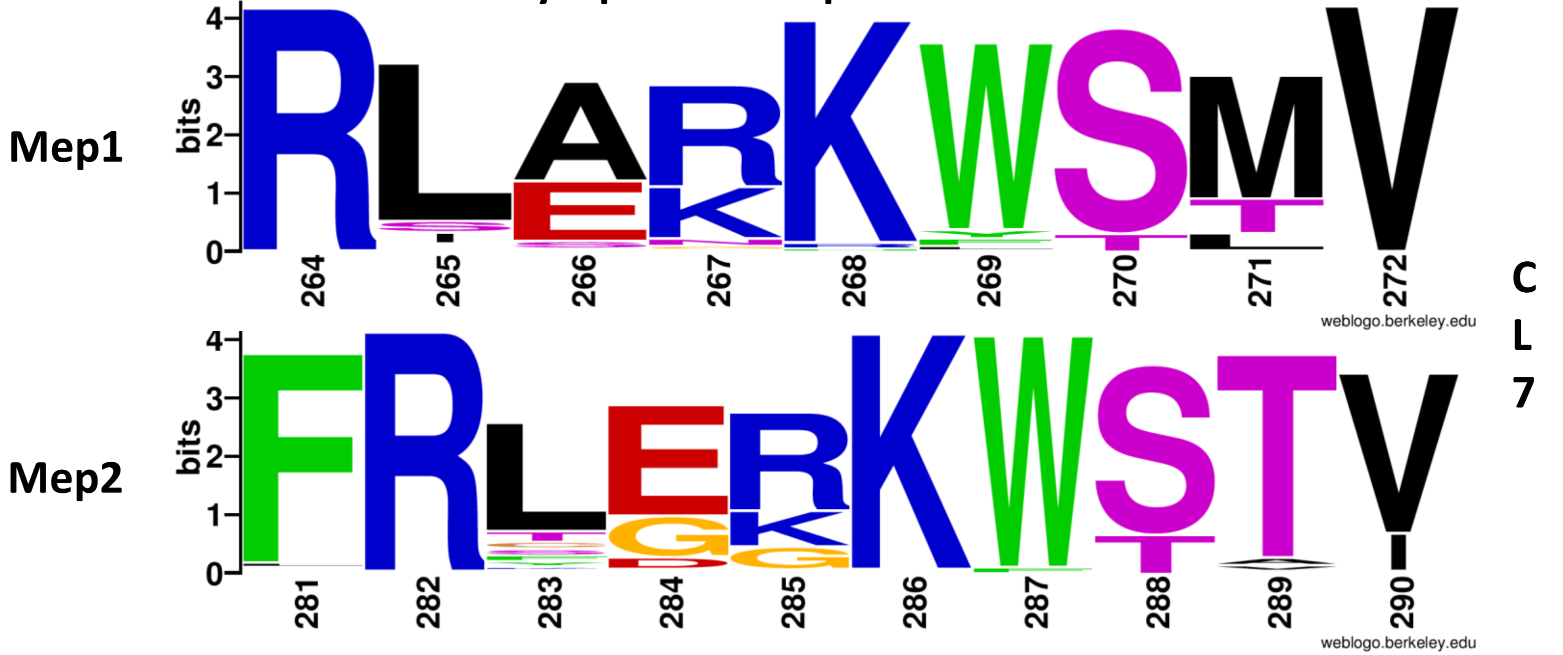

Transmembrane helix #8 (TM8)

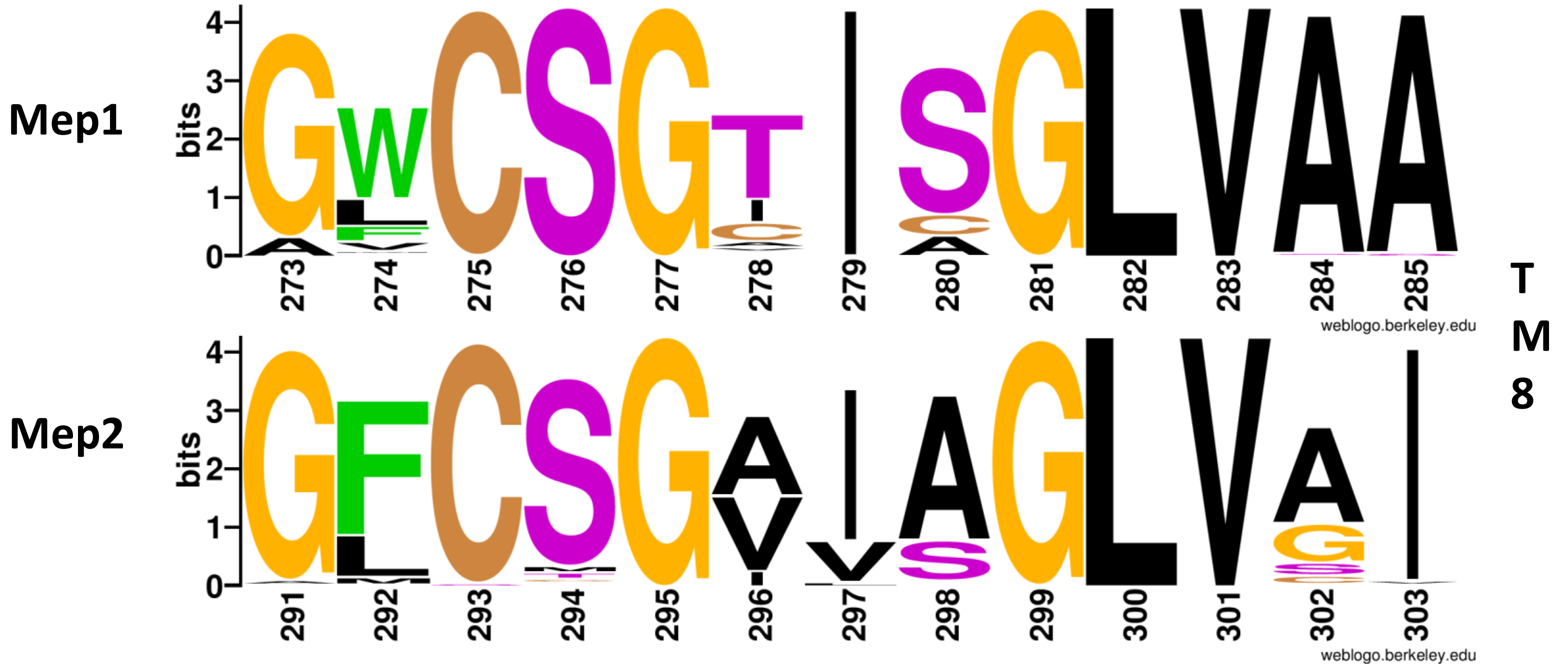

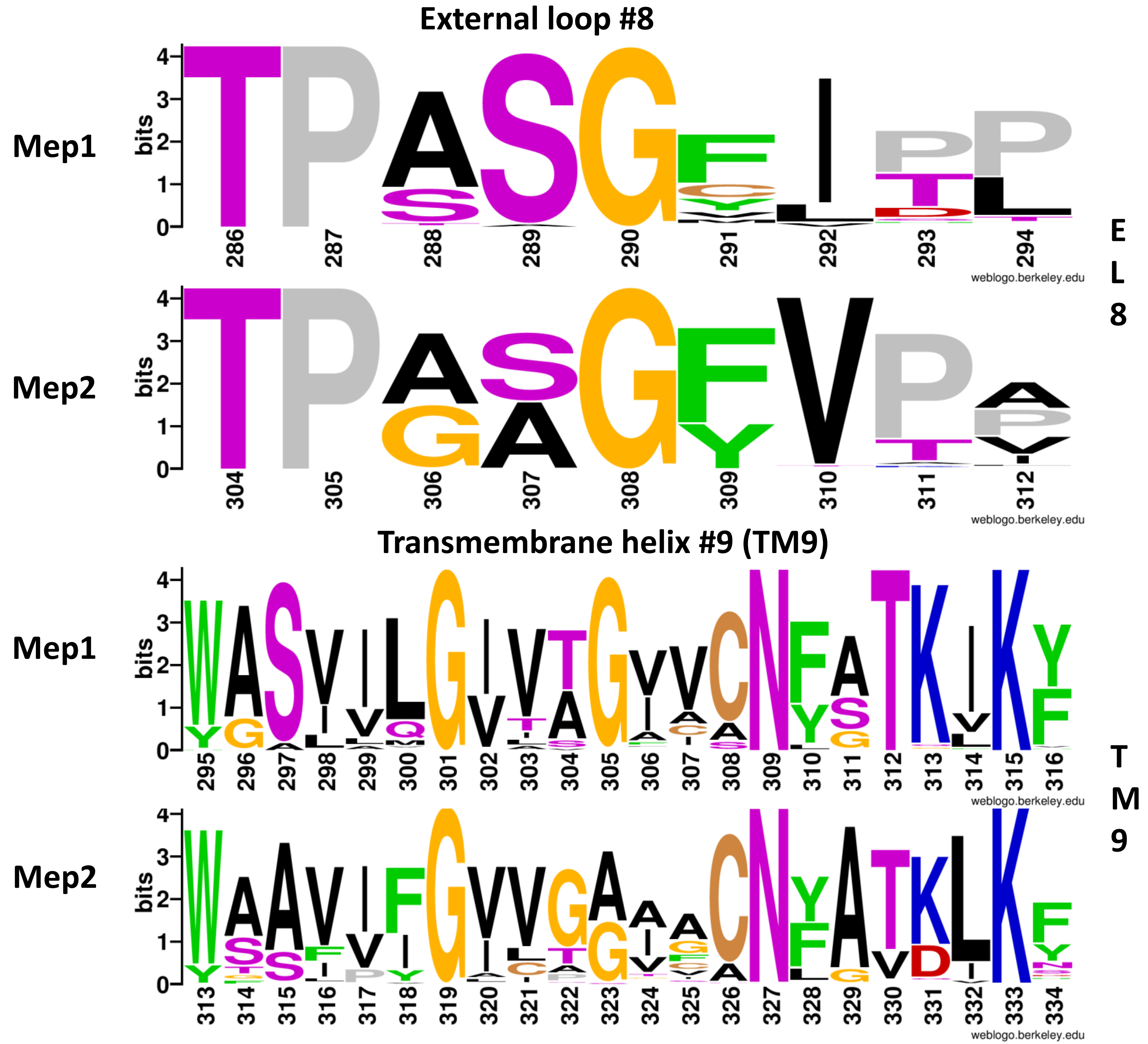
External loop #8
Mep1
Mep2
bits
weblogo.berkeley.edu
E
L
8
Transmembrane helix #9 (TM9)
T
M
9

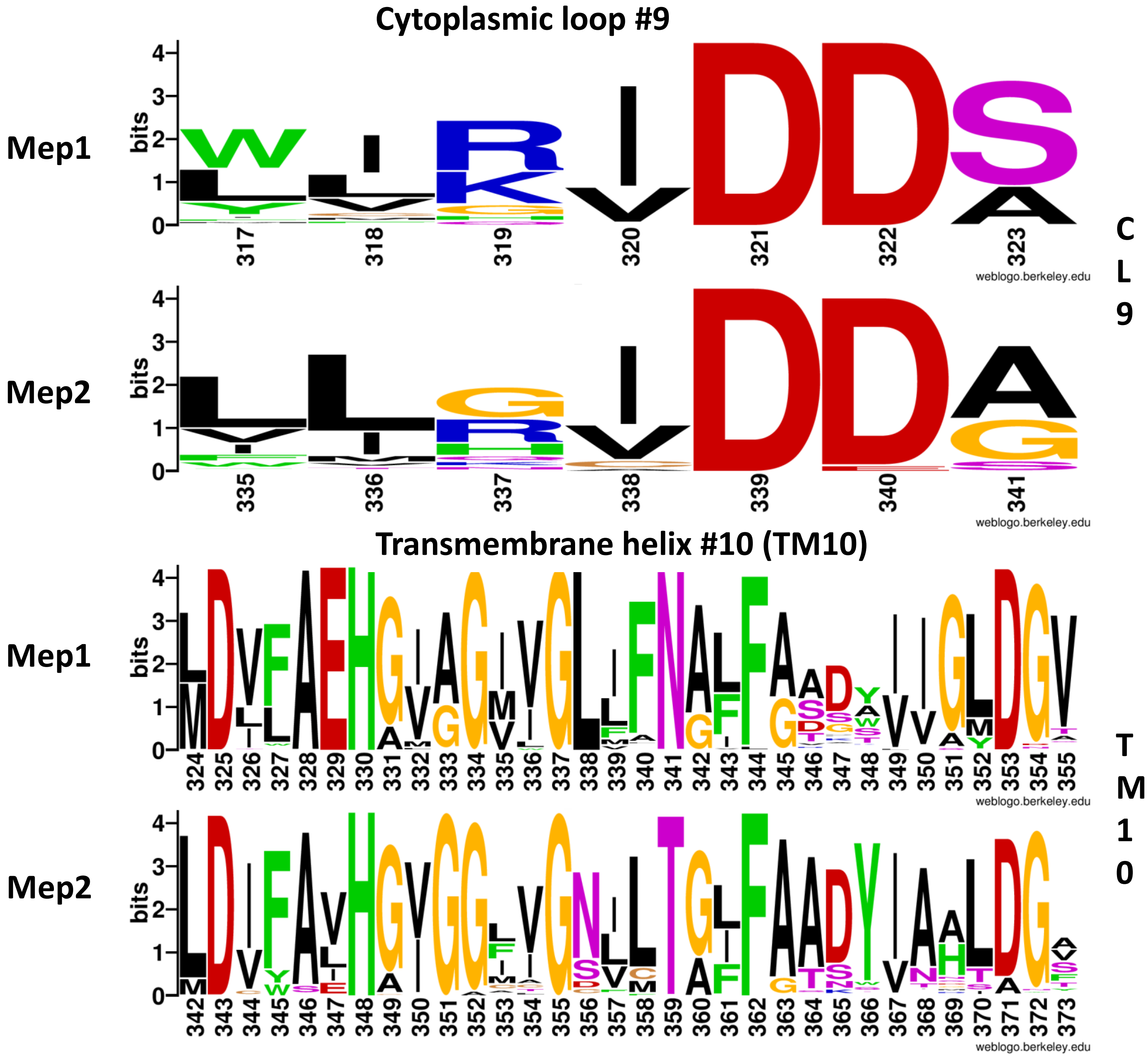
Cytoplasmic loop #9
Mep1
Mep2
CL9
Transmembrane helix #10 (TM10)
Mep1
Mep2
TM10
bits
weblogo.berkeley.edu

External loop #10

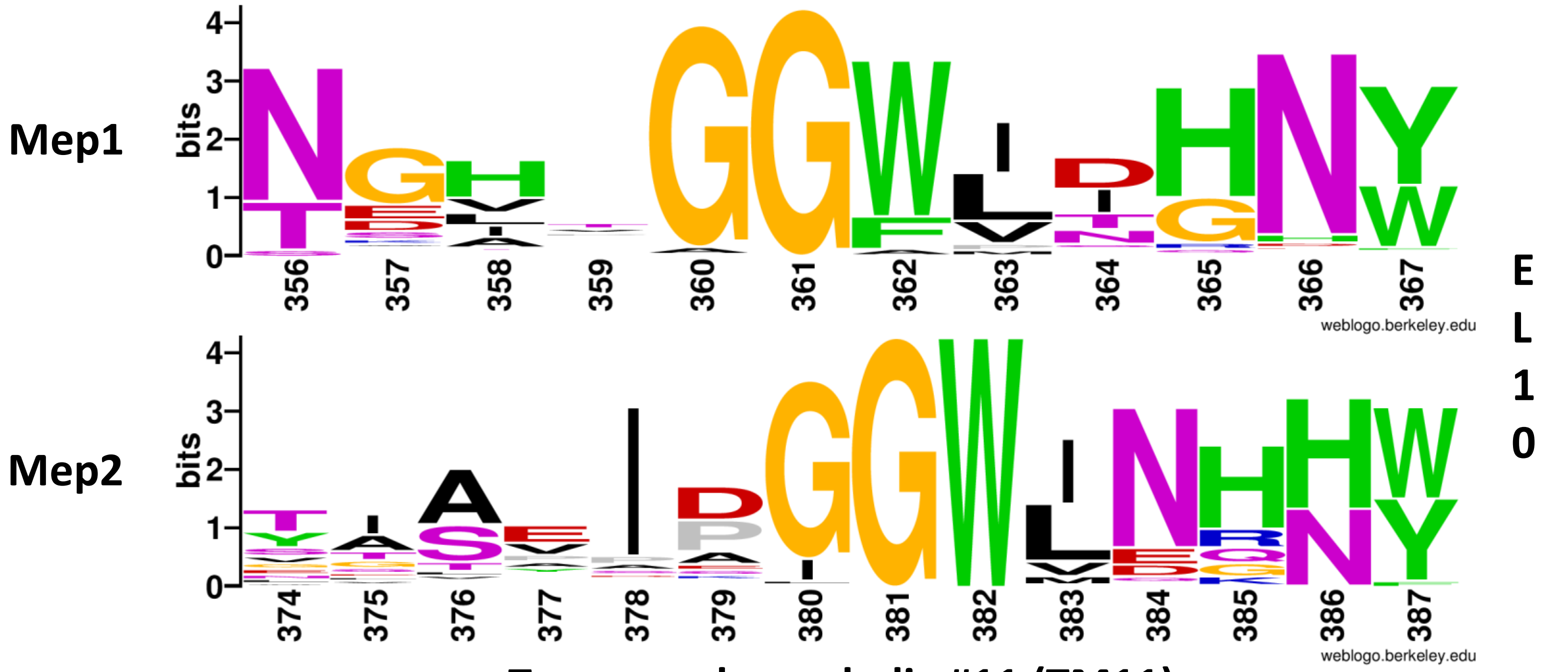

Transmembrane helix #11 (TM11)

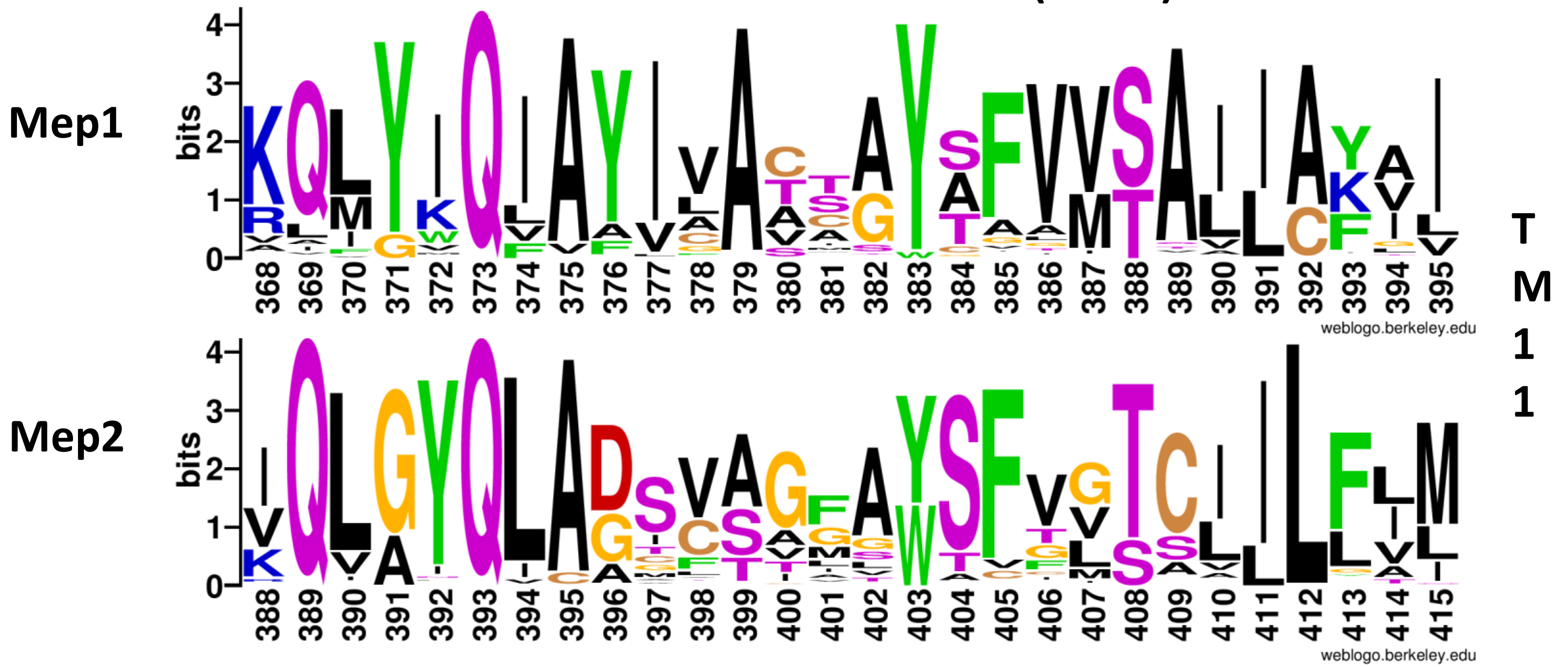

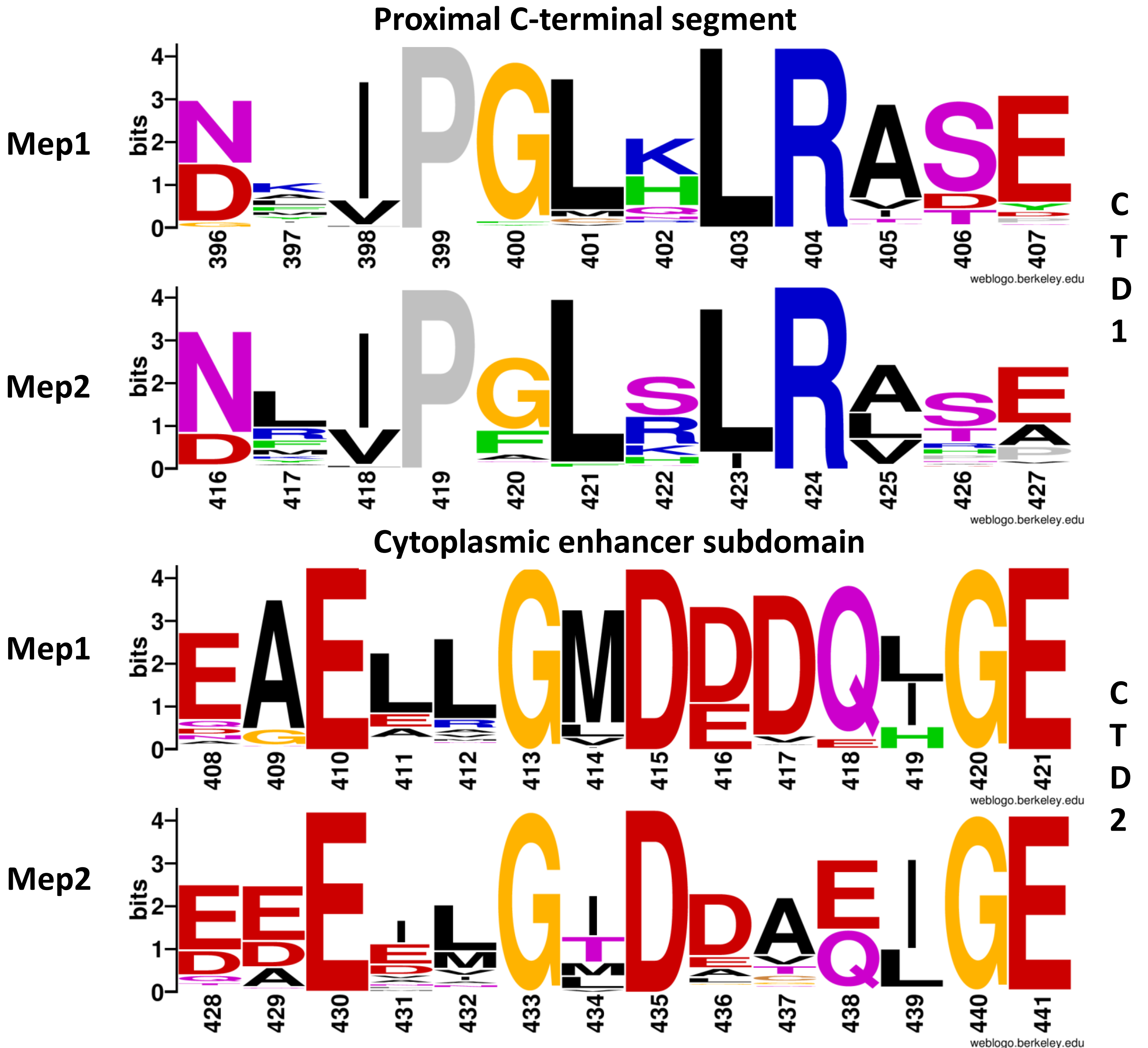

Proximal C-terminal segment
Mep1
Mep2
CTD1
Cytoplasmic enhancer subdomain
Mep1
Mep2
CTD2
bits
weblogo.berkeley.edu

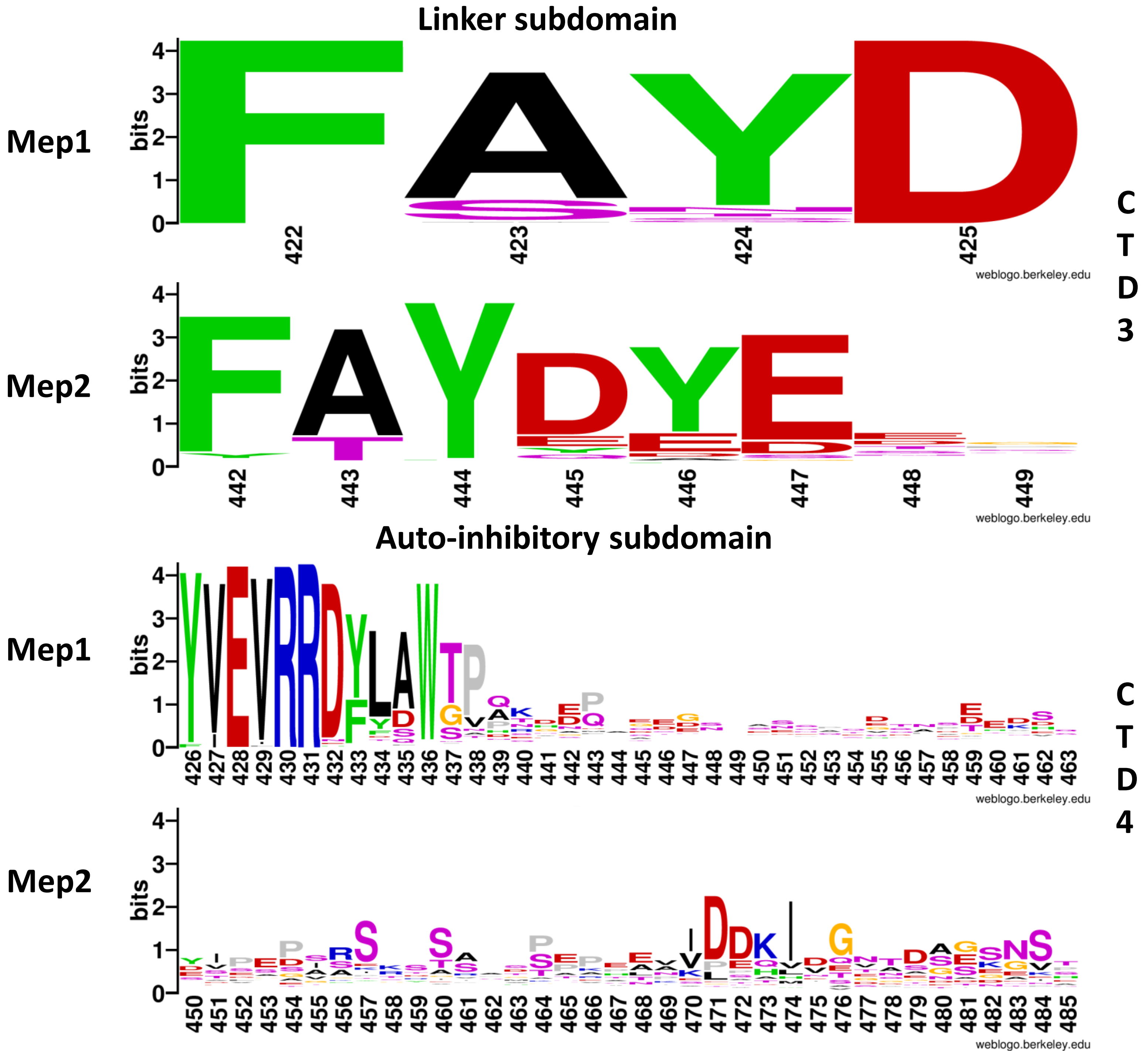
Linker subdomain
Mep1
Mep2
CTD3
Auto-inhibitory subdomain
Mep1
Mep2
CTD4
bits
weblogo.berkeley.edu

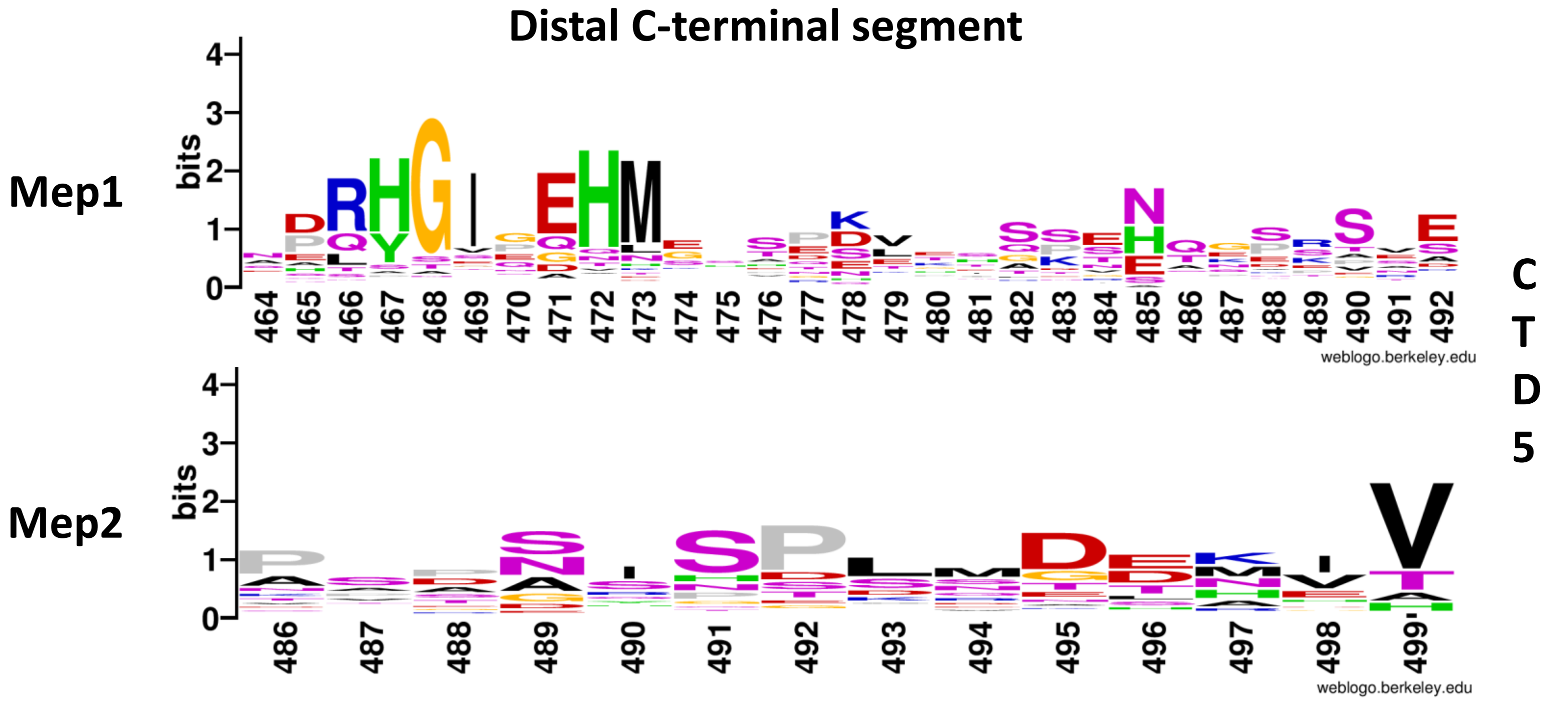
Distal C-terminal segment
Mep1
Mep2
bits
0
1
2
3
4
464 465 466 467 468 469 470 471 472 473 474 475 476 477 478 479 480 481 482 483 484 485 486 487 488 489 490 491 492
486 487 488 489 490 491 492 493 494 495 496 497 498 499
weblogo.berkeley.edu
C
T
D
5
