# Supplementary material for: Yeast filamentation signaling is connected to a specific substrate translocation mechanism of the Mep2 transceptor
Source: PLoS Genet. 2020 Feb 18;16(2):e1008634. doi: 10.1371/journal.pgen.1008634 (PMC7048316; doi:10.1371/journal.pgen.1008634)
Supplement: S1 Table — The localization of mutations in the Mep2 topology is indicated (CTD, cytoplasmic C-terminal domain; TM, transmembrane domain; EL, extracellular loop). The growth on SLAD and SHAD was determined from the growth tests shown in Figs 2A, 3A, 4B, 9A and S1A. The ammonium removal capacity was determined from Figs 1C and 4C–4E. The pseudohyphal capacity was determined from Figs 2B, 3B, 3E, 4A, 9B and S1B. Symbols for growth: ++, strong; +, intermediate or like wild-type; +/-, weak; -, absence of growth. Symbols for ammonium transport: ++, very strong; +, intermediate or like wild-type; +/-, low; -, very low. Symbols for pseudohyphal growth: ++, very strong; +, visible; +/-, weak; -, absence. ND, not determined. (PDF) [file pgen.1008634.s003.pdf]

	Localization	**Growth on low ammonium** (SLAD, 7 days)	**Growth on high ammonium** (SHAD, 7 days)	**Ammonium transport**	**Pseudohyphal growth**
**Triple-*mep*Δ**		-	-	-	-
Mep2		+	+	+	+
Mep1		+	+	ND	-
$Mep2^{S426stop}$	CTD	+/-	+/-	-	-
$Mep2^{C\Delta428\text{-}431}$	CTD2	+/-	+/-	+/-	-
$Mep2^{C\Delta428\text{-}449}$	CTD2-CTD3	+/-	+/-	+/-	-
$Mep2^{C\Delta434\text{-}449}$	CTD2-CTD3	+/-	+/-	+/-	-
$Mep2^{C\Delta434\text{-}485}$	CTD2-CTD5	+/-	+/-	+/-	-
$Mep2^{C\Delta442\text{-}449}$	CTD3	+	+	+	+
$Mep2^{C\Delta442\text{-}485}$	CTD3-CTD5	+	+	+	+
$Mep2^{C\Delta450\text{-}485}$	CTD4-CTD5	+	++	++	++
$Mep2^{C\Delta469\text{-}485}$	CTD4-CTD5	+	+	+	+
$Mep2^{D186N}$	EL4	-	-	ND	-
$Mep2^{S457D}$	CTD4	+	+	ND	+
$Mep2^{D186N, S457D}$	EL4, CTD4	-	-	ND	-
$Mep2^{H199Y}$	TM5	+	+	++	++
$Mep2^{H199Y, S426stop}$	TM5	+	+	++	+
$Mep2^{G349C}$	TM10	+	++	++	+
$Mep2^{G349C, S426stop}$	TM10	+	+	+	-
$Mep2^{H194E}$	TM5	+	+	++	-
$Mep2^{H348A}$	TM10	+/-	+	-	-
YCpCaMep2		-	+/-	ND	-
$YCpCaMep2^{H188E}$	TM5	-	+/-	ND	-
YEpCaMep2		+	+	ND	+

YEpCaMep2^{H188E}	TM5	+/-	+	ND	-
***mep2*Δ**		+	+	ND	-
Mep2		+	+	ND	+
Mep2^{D186N}	EL4	+	+	ND	-
Mep2^{S457D}	CTD4	+	+	ND	+
Mep2$^{D186N, S457D}$	EL4, CTD4	+	+	ND	-
Mep2^{S426stop}	CTD	+	+	ND	-
Mep2$^{H199Y, S426stop}$	TM5	+	+	ND	+/-
Mep2$^{C\Delta 428\text{-}431}$	CTD2	+	+	ND	-
Mep2$^{C\Delta 428\text{-}449}$	CTD2-CTD3	+	+	ND	-
Mep2$^{C\Delta 434\text{-}449}$	CTD2-CTD3	+	+	ND	-
Mep2$^{C\Delta 434\text{-}485}$	CTD2-CTD5	+	+	ND	-
Mep2^{H194E}	TM5	+	+	ND	-
Mep2^{H348A}	TM10	+	+	ND	-
**Triple-*mep*Δ *npr1-1***		-	-	-	-
Mep2		-	-	-	-
Mep2^{S426stop}	CTD	+/-	+/-	ND	-
Mep2$^{C\Delta 428\text{-}431}$	CTD2	+/-	+	+/-	+/-
Mep2$^{C\Delta 428\text{-}449}$	CTD2-CTD3	+/-	+	+	+
Mep2$^{C\Delta 434\text{-}449}$	CTD2-CTD3	+	+	+	+
Mep2$^{C\Delta 434\text{-}485}$	CTD2-CTD5	+	+	+	+
Mep2$^{C\Delta 442\text{-}449}$	CTD3	+	+	+	++
Mep2$^{C\Delta 442\text{-}485}$	CTD3-CTD5	+	+	+	++
Mep2$^{C\Delta 450\text{-}485}$	CTD4-CTD5	+	++	++	++
Mep2$^{C\Delta 469\text{-}485}$	CTD4-CTD5	+/-	+/-	+/-	-
Mep2^{H199Y}	TM5	+	+	ND	++
Mep2$^{H199Y, S426stop}$	TM5	+	+	ND	+

Mep2^{G349C}	TM10	+	+	ND	++
Mep2$^{G349C, S426stop}$	TM10	+	+	ND	+/-
Mep2^{H194E}	TM5	+	+	ND	-
Mep2^{H348A}	TM10	-	-	ND	-
**Triple-*mep*Δ *psr1*Δ *psr2*Δ**		-	-	ND	-
Mep2		+	+	ND	+
Mep2^{S457D}	CTD4	+	+	ND	+

Symbols for growth: ++, strong; +, intermediate or like wild-type; +/-, weak; -, absence of growth. Symbols for ammonium transport: ++, very strong; +, intermediate or like wild-type; +/-, low ; -, very low. Symbols for pseudohyphal growth: ++, very strong; +, visible; +/-, weak; -, absence. ND, not determined.
