# Supplementary material for: Yeast filamentation signaling is connected to a specific substrate translocation mechanism of the Mep2 transceptor
Source: PLoS Genet. 2020 Feb 18;16(2):e1008634. doi: 10.1371/journal.pgen.1008634 (PMC7048316; doi:10.1371/journal.pgen.1008634)
Supplement: S2 Table — The Protein stability changes (ΔΔG) were predicted using 7 different programs. The ΔΔG values are indicated in kcal/mol and were categorized into “Highly destabilizing” (ΔΔG ≤ -2 kcal/mol), “Destabilizing” (-2 kcal/mol < ΔΔG < -0.5 kcal/mol), “Neutral” (-0.5 kcal/mol ≤ ΔΔG ≤ +0.5 kcal/mol), “Stabilizing” (+0.5 kcal/mol < ΔΔG < +2 kcal/mol) and “Highly stabilizing” (ΔΔG ≥ +2 kcal/mol). A prediction consensus was generated by averaging the 7 results. The ΔΔG values for the consensus were indicated as mean ± SEM, where SEM is the standard error of the mean. (PDF) [file pgen.1008634.s004.pdf]

	H194E	H199Y	H348A	G349C
**INPS3D**	Destabilizing<br>-0.99	Stabilizing<br>+1.05	Destabilizing<br>-0.76	Neutral<br>-0.24
**mCSH**	Highly destabilizing<br>-2.20	Neutral<br>-0.22	Destabilizing<br>-1.81	Highly destabilizing<br>-2.08
**I-mutant3.0**	Destabilizing<br>-0.65	Neutral<br>-0.39	Destabilizing<br>-0.73	Destabilizing<br>-0.58
**ENCom**	Neutral<br>-0.28	Neutral<br>+0.30	Destabilizing<br>-0.60	Neutral<br>+0.11
**SDM**	Neutral<br>-0.43	Neutral<br>-0.39	Neutral<br>-0.05	Destabilizing<br>-0.75
**DUET**	Destabilizing<br>-2.00	Neutral<br>-0.07	Destabilizing<br>-1.63	Highly destabilizing<br>-2.05
**Dynat-Mut**	Stabilizing<br>+0.90	Stabilizing<br>+0.76	Destabilizing<br>-1.76	Destabilizing<br>-0.67
**Consensus**	**Destabilizing**<br>**-0.81 ± 0.37**	**Neutral**<br>**+0.15 ± 0.20**	**Destabilizing**<br>**-1.05 ± 0.24**	**Destabilizing**<br>**-0.89 ± 0.33**
