# Supplementary material for: Yeast filamentation signaling is connected to a specific substrate translocation mechanism of the Mep2 transceptor
Source: PLoS Genet. 2020 Feb 18;16(2):e1008634. doi: 10.1371/journal.pgen.1008634 (PMC7048316; doi:10.1371/journal.pgen.1008634)
Supplement: S3 Table — (PDF) [file pgen.1008634.s005.pdf]

Strain	Genotype	Reference
23344c	*ura3*	Lab collection
30788a	*npr1Δ ura3*	[1]
31019b	*mep1Δ mep2Δ::LEU2 mep3Δ::KanMX2 ura3*	[2]
31018b	*mep2Δ::LEU2 mep3Δ::KanMX2 ura3*	[2]
31022a	*mep1Δ mep3Δ::KanMX2 ura3*	[2]
ZAM38	*mep1Δ/mep1Δmep2Δ::LEU2/mep2Δ::LEU2 mep3Δ::KanMX2/mep3Δ::KanMX2 ura3/ura3*	[3]
ZAB2	*mep2Δ::LEU2/mep2Δ::LEU2 ura3/ura3*	This study
ZMB058	*mep1Δ/mep1Δ mep2Δ::LEU2/mep2Δ::LEU2 mep3Δ::KanMX2/mep3Δ::KanMX2 npr1-1/npr1-1 ura3/ura3*	[4]
ZAB1	*mep1Δ/mep1Δ mep2Δ::LEU2/mep2Δ::LEU2 mep3Δ::KanMX2/mep3Δ::KanMX2 psr1Δ::Phleo/psr1Δ::Phleo psr2Δ::Hyg/psr2Δ::Hyg ura3/ura3*	This study

1. De Craene JO, Soetens O, Andre B. The Npr1 kinase controls biosynthetic and endocytic sorting of the yeast Gap1 permease. The Journal of biological chemistry. 2001;276(47):43939-48. doi: 10.1074/jbc.M102944200. PubMed PMID: 11500493.

2. Marini AM, Soussi-Boudekou S, Vissers S, Andre B. A family of ammonium transporters in Saccharomyces cerevisiae. Molecular and cellular biology. 1997;17(8):4282-93. PubMed PMID: 9234685; PubMed Central PMCID: PMC232281.

3. Marini AM, Boeckstaens M, Benjelloun F, Cherif-Zahar B, Andre B. Structural involvement in substrate recognition of an essential aspartate residue conserved in Mep/Amt and Rh-type ammonium transporters. Current genetics. 2006;49(6):364-74. doi: 10.1007/s00294-006-0062-5. PubMed PMID: 16477434.

4. Boeckstaens M, Andre B, Marini AM. The yeast ammonium transport protein Mep2 and its positive regulator, the Npr1 kinase, play an important role in normal and pseudohyphal growth on various nitrogen media through retrieval of excreted ammonium. Molecular microbiology. 2007;64(2):534-46. doi: 10.1111/j.1365-2958.2007.05681.x. PubMed PMID: 17493133.
