# Supplementary material for: Yeast filamentation signaling is connected to a specific substrate translocation mechanism of the Mep2 transceptor
Source: PLoS Genet. 2020 Feb 18;16(2):e1008634. doi: 10.1371/journal.pgen.1008634 (PMC7048316; doi:10.1371/journal.pgen.1008634)
Supplement: S4 Table — (PDF) [file pgen.1008634.s006.pdf]

Plasmid	Description	Reference
**Low-copy-number**		
YCpFL38	CEN-ARS URA3	[1]
YCpMep2	YCpFL38 MEP2	[2]
YCpMep1	YCpFL38 MEP1	[3]
pGAL1Mep2-GFP	p416 GAL1-MEP2 $(GA)_5$-GFP	[4]
pMep2-pHluorin	p416 PROM MEP2-MEP2 $(GA)_5$-pHluorin	This study
pMep2^{D186N}-pHluorin	p416 PROM MEP2-MEP2^{D186N} $(GA)_5$-pHluorin	This study
pMep2^{H194E}-pHluorin	p416 PROM MEP2-MEP2^{H194E} $(GA)_5$-pHluorin	This study
YCpMep2^{H194E}	YCpFL38 MEP2^{H194E}	[5]
YCpMep2^{H199Y}	YCpFL38MEP2^{H199Y}	This study
YCpMep2^{H199Y,S426stop}	YCpFL38 MEP2^{H199Y,S426stop}	This study
YCpMep2^{D186N}	YCpFL38 MEP2^{D186N}	This study
YCpMep2^{S457D}	YCpFL38MEP2^{S457D}	This study
YCpMep2^{D186N,S475D}	YCpFL38MEP2^{D186N,S475D}	This study
YCpMep2^{S426stop}	YCpFL38 MEP2^{S426stop}	[4]
YCpMep2$^{C\Delta428\text{-}431}$	YCpFL38 MEP2$^{C\Delta428\text{-}431}$	[6]
YCpMep2$^{C\Delta428\text{-}449}$	YCpFL38 MEP2$^{C\Delta428\text{-}449}$	[6]
YCpMep2$^{C\Delta434\text{-}449}$	YCpFL38 MEP2$^{C\Delta434\text{-}449}$	[6]
YCpMep2$^{C\Delta442\text{-}449}$	YCpFL38 MEP2$^{C\Delta442\text{-}449}$	[6]
YCpMep2$^{C\Delta434\text{-}485}$	YCpFL38 MEP2$^{C\Delta434\text{-}485}$	[6]
YCpMep2$^{C\Delta442\text{-}485}$	YCpFL38 MEP2$^{C\Delta442\text{-}485}$	[6]
YCpMep2$^{C\Delta450\text{-}485}$	YCpFL38 MEP2$^{C\Delta450\text{-}485}$	[6]
YCpMep2$^{C\Delta469\text{-}485}$	YCpFL38 MEP2$^{C\Delta469\text{-}485}$	[6]
YCpMep2^{G349C}	YCpFL38 MEP2^{G349C}	[4]
YCpMep2$^{G349C, S426stop}$	YCpFL38 MEP2$^{G349C, S426stop}$	This study
YCpMep2^{H348A}	YCpFL38 MEP2^{H348A}	[4]
YCpCaMep2	YCpFL38 PROM ScMEP2-CaMEP2-TERM ScMEP2	This study
YCpCaMep2^{H188E}	YCpFL38 PROM ScMEP2-CaMEP2^{H188E}-TERM ScMEP2	This study
**High-copy-number**		
YEpFL44	2μ URA3	[1]
YEpMep1	YEpFL44 MEP1	[7]
pHluorin	pYES-pHluorin	[8]
pOO2	pOO2	[9]
pOOMep1	pOO2 MEP1	This study
pOOMep2	pOO2 MEP2	This study
pOOMep2^{H194E}	pOO2 MEP2^{H194E}	This study
pOOMep2^{S457D}	pOO2 MEP2^{S457D}	This study
pOOMep2$^{H194E, S457D}$	pOO2 MEP2$^{H194E, S457D}$	This study
pOOMep2-GFP	pOO2 MEP2 $(GA)_5$-GFP	This study
pOOMep2^{N4Q}-GFP	pOO2 MEP2^{N4Q} $(GA)_5$-GFP	This study
pOOMep2^{H194E}-GFP	pOO2 MEP2^{H194E} $(GA)_5$-GFP	This study
pOOMep2^{S457D}-GFP	pOO2 MEP2^{S457D} $(GA)_5$-GFP	This study
YEpCaMep2	YEpFL44 PROM ScMEP2-CaMEP2-TERM ScMEP2	This study
YEpCaMep2^{H188E}	YEpFL44 PROM ScMEP2-CaMEP2^{H188E}-TERM ScMEP2	This study

1. Bonneaud N, Ozier-Kalogeropoulos O, Li GY, Labouesse M, Minvielle-Sebastia L, Lacroute F. A family of low and high copy replicative, integrative and single-stranded S. cerevisiae/E. coli shuttle vectors. Yeast. 1991;7(6):609-15. doi: 10.1002/yea.320070609. PubMed PMID: 1767589.

2. Marini AM, Soussi-Boudekou S, Vissers S, Andre B. A family of ammonium transporters in Saccharomyces cerevisiae. Molecular and cellular biology. 1997;17(8):4282-93. PubMed PMID: 9234685; PubMed Central PMCID: PMC232281.

3. Marini AM, Vissers S, Urrestarazu A, Andre B. Cloning and expression of the MEP1 gene encoding an ammonium transporter in Saccharomyces cerevisiae. The EMBO journal. 1994;13(15):3456-63. PubMed PMID: 8062822; PubMed Central PMCID: PMC395248.

4. Boeckstaens M, Llinares E, Van Vooren P, Marini AM. The TORC1 effector kinase Npr1 fine tunes the inherent activity of the Mep2 ammonium transport protein. Nature communications. 2014;5:3101. doi: 10.1038/ncomms4101. PubMed PMID: 24476960.

5. Boeckstaens M, Andre B, Marini AM. Distinct transport mechanisms in yeast ammonium transport/sensor proteins of the Mep/Amt/Rh family and impact on filamentation. The Journal of biological chemistry. 2008;283(31):21362-70. doi: 10.1074/jbc.M801467200. PubMed PMID: 18508774.

6. Van Nuland A, Vandormael P, Donaton M, Alenquer M, Lourenco A, Quintino E, et al. Ammonium permease-based sensing mechanism for rapid ammonium activation of the protein kinase A pathway in yeast. Molecular microbiology. 2006;59(5):1485-505. doi: 10.1111/j.1365-2958.2005.05043.x. PubMed PMID: 16468990.

7. Boeckstaens M, Merhi A, Llinares E, Van Vooren P, Springael JY, Wintjens R, et al. Identification of a Novel Regulatory Mechanism of Nutrient Transport Controlled by TORC1-Npr1-Amu1/Par32. PLoS genetics. 2015;11(7):e1005382. doi: 10.1371/journal.pgen.1005382. PubMed PMID: 26172854; PubMed Central PMCID: PMC4501750.

8. Orij R, Postmus J, Ter Beek A, Brul S, Smits GJ. In vivo measurement of cytosolic and mitochondrial pH using a pH-sensitive GFP derivative in Saccharomyces cerevisiae reveals a relation between intracellular pH and growth. Microbiology. 2009;155(Pt 1):268-78. doi: 10.1099/mic.0.022038-0. PubMed PMID: 19118367.

9. Ludewig U, von Wiren N, Frommer WB. Uniport of NH4+ by the root hair plasma membrane ammonium transporter LeAMT1;1. The Journal of biological chemistry. 2002;277(16):13548-55. doi: 10.1074/jbc.M200739200. PubMed PMID: 11821433.
