# Supplementary material for: Yeast filamentation signaling is connected to a specific substrate translocation mechanism of the Mep2 transceptor
Source: PLoS Genet. 2020 Feb 18;16(2):e1008634. doi: 10.1371/journal.pgen.1008634 (PMC7048316; doi:10.1371/journal.pgen.1008634)
Supplement: Attachment — Submitted filename: Review_PLOS_Gen_FINAL_3.docx [file pgen.1008634.s007.docx]

Reviewer #1: In this thought-provoking manuscript, Brito, et al., examine the transport and signaling mechanisms of the Mep2 ammonium transceptor in yeast. They show that transport is required for signaling and that the cytoplasmic C-terminal domain, previously thought to be required for the signaling function, is dispensable in certain circumstances. The mutational studies are extensive, based on previously characterized sequences combined with some new structural analysis. Interestingly, the genetic experiments are coupled with measures of transport and cytoplasmic pH (in yeast) and electrochemical current (in Xenopus). These support a difference in transport mechanism between the
signaling Mep2 and non-signaling Mep1 proteins, concluding that Mep1 transports the ammonia molecule with its proton and Mep2 beings in only the proton.

These are intriguing conclusions based on solid and rigorous experiments. They do not identify the elusive signaling mechanism, but the paper does lead to new directions for understanding it. The
electrophysiology in Xenopus is particularly persuasive. The subject matter and approach are appropriate for PLoS Genetics. A few, mostly minor, comments are below.

1. The weakest experimental aspect of the paper is the measurements of cytosolic pH. There are believable differences between Mep1 and Mep2, but they are quite small. This is likely because of the proton pumps in the plasma and vacuolar membranes. Perhaps if these were inhibited, pharmacologically or genetically, the differences would be enhanced and the confounding pH recovery phase reduced.

As anticipated by Reviewer 1, the differences in pHc recorded upon substrate translocation via Mep1 and Mep2 are small and this likely partially accounts for the rapid counteracting action of Pma1, the plasma membrane proton ATPase. Besides this, large pHc differences are not expected considering that we are following the activity of secondary transport proteins and that the translocated protons or buffering ammonia molecules need to diffuse through the cytosol to be reported by a difference in fluorescence emission by cytosolic pHluorin. The experiment proposed by the Reviewer appears sound at first sight but is difficult to design and its results would be hard to interpret. There are no specific Pma1 inhibitors described so far. *PMA1* is an essential gene. Pma1 is required for growth, it is responsible for the plasma membrane electrochemical gradient supporting numerous transport events. Even if we were able to reduce Pma1 activity, this would in first instance reduce/inhibit Mep1 and Mep2 transport activities. We have shown that the transport activities of Mep1 and Mep2 are sharply reduced in the presence of the CCCP protonophore, a condition removing the proton gradient (Boeckstaens et al., 2008). Similarly, Mep proteins activity is lost upon glucose removal and consequent reduction in the ATP pool that feeds Pma1 activity. Even if we could manage to reduce Pma1 activity, Mep proteins would in turn show a reduced activity in these conditions, thereby making hard to interpret the influence of Pma1 inhibition on the pH change induced by transport activity of the Meps.

To try to address the question of this Reviewer, we designed an experiment to assess the submembrane, local, consequence of substrate transport via Mep proteins by directly fusing pHluorin at their C-terminal extremity. The fluorescence emission by Mep1-pHluorin was too low to serve as a reporter of translocation activity, most likely due to the low expression level of the chimera reached with the natural *MEP1* promoter. By contrast, Mep2-pHluorin expressed under the control of the natural *MEP2* promoter was localized at the plasma membrane and produced a specific emission signal. In cells expressing the inactive Mep2^D186N^-pHluorin, addition of ammonium (2 or 0.5 mM) had no clear impact on the submembrane pH, consistent with the inability of Mep2^D186N^ to recognize and transport ammonium (Fig. 7B). In cells expressing Mep2-pHluorin, ammonium addition, either at 2 or 0.5 mM, induced an initial acidification phase followed by a rapid alkalinisation phase (Fig. 7A). Of note, in cells expressing Mep2^H194E^-pHluorin, addition of 2 or 0.5 mM ammonium induced a pronounced transient drop of pH followed by a pH recovery phase (Fig. 7C). The drop reached 0.1 unit and was even bigger than the one observed for cytosolic pH in cells expressing Mep1 (Fig. 6C). This pH change does not sound so small considering that a process like apoptosis is induced by a delta pH of 0.3-0.4 (Srivastava *et al.*, 2007).

We have included the Mep2-pHluorin experiments in the revised manuscript (Figure 7). We also discussed the point of the small Mep-dependent pH change in the discussion section (lines 608-620).

2. The genetic experiments are thorough, but it is a bit confusing to keep track of all the mutants and what they are. A cartoon of the linear protein sequence, with the TMs, the enhancer, AI domains, and the position of the point mutants would be very helpful. Perhaps this could be added at the top of Fig. 1?

We agree with the Reviewer. We added a detailed cartoon as new Figure 1, representing the topology of the Mep2 protein with imbedded sequence and highlighted key residues.

3. Are they sure that the Mep2 enhancer domain mutants, which have reduced transport and signaling activity are stably expressed? A Western would be confirmatory (was this not done because the C-terminal antibody does not recognize these mutants well?).

We agree with the Reviewer that it could be informative to look at the stability of the C-terminal Mep2 enhancer variants. The lab has formerly developed two anti-Mep2 peptides polyclonal antibodies. The first ones target epitopes in the C-terminal domain encompassing residues 450-499 (Marini and André 2000). They can’t be used to detect all C-terminal variants and in addition they could also be sensitive to phosphorylation events occurring on epitopes in this Mep2 domain. The second ones target a short peptide including phosphorylated S457 (Boeckstaens et al., 2014) and can mainly be used to address the phosphorylated state of S457.

Using the available anti-“450-499” polyclonal antibodies, the abundance of 3 of the 4 enhancer domain variants can nevertheless be assessed. These variants were detectable by western blot although they appeared slightly less abundant than native Mep2. We added this result in the manuscript (Fig. 2C).

We also tried to obtain new polyclonal anti-Mep2 antibodies targeting small specific peptides, either in the loop separating TM5 and TM6, either between T10 and TM11. We received the purified antibodies lately and are testing them. The first ones are not working in tested conditions while the second ones allow to specifically detect Mep2 but not efficiently. Upon overexposure, we succeeded in detecting Mep2 in Npr1-lacking cells grown on proline medium where the protein is likely more abundant. Variants in the enhancer, linker and autoinhibitory domains are all detectable with no major variation as shown in the figure below. We prefer not to include these data in the revised manuscript. It would require more time to fine tune the procedure and these additional data would not bring a lot more information at the end.

4. Some of the figures (especially Fig. 1) have many constructs tested and it is challenging to really sort out which line represents which mutant. A consistent color scheme would help greatly. Alternatively,
splitting this into two figures with the enhancer domain mutants in one and the others in another might help.

As suggested by several Reviewers, we split and added colors to the graphs from former Fig.1, (new Fig. 1C).

Reviewer #2: Filamentation is the critical process for fungal development and pathogenicity of many fungal pathogens. In many fungi, filamentation is triggered by nitrogen starvation and therefore Mep2,
ammonium permease, has been implicated in the process. A question remains whether Mep2 has a ‘transceptor’ activity for the filamentation process. That is to say, does Mep2 not only sense ammonium, but also transport it for filamentation?

We may precise here that Mep2 is able to transport ammonium in the filamentation conditions (Marini et al., 1997; Marini and André, 2000). Contrary to a strain deleted for the three *MEP* genes, a strain expressing only the *MEP2* gene (deleted for *MEP1* and *MEP3*) is able to grow on SLAD, in which ammonium is the sole nitrogen source provided at low concentration. Ammonium needs to be transported for growth, and filamentous growth, to occur. Whether Mep2 alternates, according to the local ammonium concentration, between a transporter conformation, only enabling to store the nitrogen source, and a signaling conformation only allowing signaling, is difficult to test.

In this paper, Brito et al. tackled this question through genetic, molecular, and structure analyses of yeast Mep2. Through domain deletion experiments of Mep2 in triple mep deletion mutant background, the authors clearly demonstrated that ammonium transport function is tightly correlated with filamentation efficiency. They showed that the phosphomimetic mutation S457D in the C-terminal autoinhibitory domain of Mep2 did not lock Mep2 as a constitutive filamentation active form and it still required substrate translocation.
Previously, it has been shown that the C-terminal domain (CTD) plays a role in tuning the transport activity of Mep2. Here the authors demonstrated that the CTD domain is not absolutely required for
filamentation induction, because the H199Y and G349C suppressor mutation in the transmembrane domains can trigger filamentation even without the CTD domain. Based on the analysis based on reported X-ray structure of ScMep2, H199 and G349, which are evolutionarily divergent and conserved
among other fungal orthologs, respectively, are facing each other. Subsequently, they observed the different impact of Mep1 and Mep2 on cytosolic pH. Supporting this, electrophysiology analyses using
heterologous expression of Mep1 and Mep2 in Xenopus oocytes demonstrated that non-filamentation signaling Mep1 mediated electrogenic transport while Mep2 mediated electroneutral substrate translocation.
Overall, this study provides very informative and valuable data for confirming the transceptor activity of Mep2 required for yeast filamentation. Here the authors clearly demonstrated that the Mep2
transceptor not only functions as ammonium transporter and a signaling receptor for filamentation, even without involvement of cytosolic partner proteins. So intracellular pH change can be a signal mediator for yeast filamentation. The manuscript is very well written and most of conclusions are nicely supported by a sophisticated set of experiments. I have the following minor comments.

[Minor comments]

Each paragraph should be indented throughout the manuscript.

We now paid attention to this point throughout the revised manuscript.

The first paragraph of the Introduction section is too long. It needs to be divided into two paragraphs (line 65?)

We agree. This paragraph has been divided into two paragraphs.

Line 76-78: The meaning of this sentence is unclear. Please clarify it with more detailed explanation.

“Of the three yeast *MEP* genes, the specific deletion of *MEP2* impairs filamentation while ammonium transport in the cells is largely unaffected, being ensured by the Mep1 and Mep3 paralogues.” We mean that a strain deleted only for the *MEP2* gene is able to grow on SLAD thanks to the ammonium transport activity of the remaining Mep1 and Mep3 proteins that ensure ammonium transport required for growth. However, this strain is unable to induce pseudohyphal growth, showing that Mep2 is specifically required for the dimorphic change. In addition, a strain deleted for *MEP1* and *MEP3* is still able to grow on SLAD thanks to the transport activity of Mep2, and also to perform filamentous growth thanks to the supposed signaling function of Mep2.

We now provide a more detailed description in the revised manuscript (lines 78-82).

Lines 132-141: A graphic illustration of each domain in the Mep2 protein (in Figure 1?) should make readers better understood. In addition, any previously known model describing how Mep2 protein works in association with Npr1 kinase could help readers too.

As suggested this and by other Reviewers, we added a graphic illustration of Mep2 and the model of Mep2 regulation by Npr1 in the new Figure 1.

It seems that only two independent experiments were performed for Figure 1B and Figure 3C experiments. For statistical analysis, at least three biological independent experiments should be performed and proper statistical analysis should be done.

We thank the Reviewer for the careful examination of the data. Experiments were repeated, and new ones performed, so that all shown kinetic measurements come now from at least 3 independent biological replicates, thereby allowing accurate statistical analysis.

The graphs in Figure 2B are very difficult to see. Please use colored lines with more distinct symbols.

As suggested by several Reviewers, we added colors to the graphs from former Fig.1B (new Fig.1C). Moreover, as suggested by one other Reviewer, we split the graphs into several ones for better reading.

Each Figure 5 graph should be separately plotted at 2 mM and 20 mM NH3, as the two data are overlapped and hard to discriminate.

We thank the Reviewer for this comment. This comment reflects a mistake in the legend and in the text. It has been corrected (lines 382-384). The condition “20 mM ammonium” has been only used in Fig.6A (former Fig.5A). The condition “2mM ammonium” is used in Fig.6A-D. In the panel A, the only one to mix the 2mM and 20mM conditions, the plots do not overlap.

Reviewer #3: This study aimed at elucidating which specific aspects of the ammonium permease Mep2, as opposed to its paralogs Mep1 and Mep3, are responsible for its ability to induce filamentous growth in response to nitrogen limitation in the budding yeast Saccharomyces cerevisiae. By testing the ability of mutated forms of Mep2 to mediate ammonium transport and promote filamentation, the authors confirmed their previous observation that the induction of filamentous growth by Mep2 generally correlates with its ammonium transport activity. The C-terminal cytoplasmic tail of Mep2 was found to be dispensable for the induction of filamentous growth by some mutated forms of the protein,
and the authors conclude that it does not represent a signaling domain. Electrophysiological studies showed that ammonium transport by Mep2 is electroneutral (i.e., it transports NH3 without H+ after deprotonation of NH4+) whereas transport by Mep1 is electrogenic (i.e., the proton is co-transported). Accordingly, the authors observed a slight increase in the cytosolic pH after ammonium addition to cells containing Mep2, which was weaker in cells producing only Mep1. The authors conclude that pH
modification is the mechanism by which Mep2 induces filamentous growth.

This is a very interesting study that addresses an unresolved question, how Mep2 and related ammonium permeases in other fungi induce morphogenesis. There are a number of issues that in my opinion affect the authors’ main conclusions and should be considered.

Comments

1) It is difficult to envisage that the very minor (less than 0.05 pH units) and presumably short-term (minutes) increase in the cytosolic pH after the addition of ammonium to the cells is the trigger for
filamentous growth, considering that the induction of filamentous growth occurs after several days of growth under limiting nitrogen conditions; one would assume that the cells maintain intracellular pH homeostasis under these conditions. Furthermore, the experiments shown in Fig. 5 were performed in the presence of 2 mM ammonium, a concentration that suppressed filamentous growth in the present study (see Fig. 1C, Fig. 2B and E, Fig. 3B). At the low concentrations of ammonium that
induce filamentation, one would expect a still smaller, if any, effect on cytosolic pH by Mep2-mediated ammonium uptake. Of note, Mep2 also induces filamentous growth at low concentrations of other nitrogen sources, and this is believed to be due to Mep2-mediated retrieval of
excreted ammonium. Under these conditions, even less extracellular ammonium will be present that can be taken up by Mep2 and affect the cytosolic pH. Although the authors also state that conformational changes in Mep2 that occur during ammonium transport are involved in signaling, the sentences at the end of the abstract, author summary, introduction, and discussion convey the message that Mep2-mediated pH changes are the key signal that induces filamentation.

We thank the Reviewer for raising all these key points. Since Mep2 is a transport protein, it is to us important to exclude that a transport-related consequence is not the leading cause of filamentation induction before to be convinced that Mep2 is a receptor. At least the data we have with ScMep2 indicate that it is difficult to separate the transport and signaling function, and when this is achieved with the ScMep2^H194E^ variant, the protein appears to show modification of the transport mechanism. In the experiment of former figure 5 (new Fig.6), we tried to have an experimental set up with controlled conditions using cells in balanced exponential phase of growth in a buffered proline medium, in conditions where we know that we can measure the activity of Mep1 and Mep2, either via ^14^C-methylammonium uptake assays or ammonium removal assays (Marini et al., 1994, 1997), so in conditions in which we are sure that if Mep activity would lead to a pH change this could be measured. But of course, considering that those proteins are able to deprotonate NH_4_^+^, the fate of the released proton might depend on the physiological state of the cells, on the electrochemical gradient that predominates in one determined environment, and thus could vary within the filamentation conditions of SLAD.

The ideal experiment would be to measure Mep2 activity and pH modifications in conditions of filamentation, so in cells growing for several days on SLAD, thus largely starved for nitrogen. This is technically very difficult to design. The *MEP2* gene, which responds to the nitrogen catabolite repression, is probably more expressed in condition of filamentation than on the proline medium used to measure pH changes. This overproduction of the protein may lead to an accentuated transport of NH_3_, leading to a more important alkalinisation. If we follow the SHAD plates for a longer period than 7 days, filamentation also occurs in a Mep2-dependent manner. Also, if Mep2 is overexpressed, or expressed under the *GAL1* promoter on SHAD, this gives rise to clear filamentation (Rutherford et al., 2008, and our unpublished data). If we consider that Mep2 leaves the proton in the periplasm as part of the transport mechanism, this could also constitute an advantage for cells quasi-starved for nitrogen. This could contribute to maintain an electrochemical gradient in turn favoring the transport of other surrounding molecules.

As detailed in the response to point 1 of Reviewer 1 and point 4 of this Reviewer, we designed an experiment to assess the submembrane, local, consequence of substrate transport via Mep proteins by directly fusing pHluorin at their C-terminal extremity. In cells expressing Mep2-pHluorin, ammonium addition, either at 2 or 0.5 mM, induced an initial acidification phase followed by a rapid alkalinisation phase (Fig.7A). Note here that using 0.5 mM ammonium, so closer to the initial ammonium concentration of SLAD medium, produced a similar effect compared to 2mM. This could be explained by a regulation of the activity of the Mep in function of the ammonium concentrations. Our previous results have already suggested that Mep proteins are inactivated by high ammonium concentrations (Boeckstaens *et al.*, 2014 and 2015). Of note, in cells expressing Mep2^H194E^-pHluorin, addition of 2 or 0.5 mM ammonium induced a pronounced transient drop of pH followed by a pH recovery phase (Fig.7C). The drop reached 0.1 unit and was even bigger than the one observed for cytosolic pH in cells expressing Mep1 (Fig.6C). This pH change could be meaningful considering that a process like apoptosis is induced by a delta pH of 0.3-0.4 (Srivastava *et al.*, 2007).

An important message of our manuscript is that Mep1 and Mep2 have the ability to transport ammonium via different transport mechanisms. These results are also sustained by electrophysiological data. Our data show a correlation between the transport mechanism (associated to a pH change) and the capacity to induce filamentation.

We have included the Mep2-pHluorin experiments in the revised manuscript (Figure 7). We also discussed the point of the small Mep-dependent pH change in the discussion section (lines 608-620). We have revised the text to temperate the conclusion.

2) The presentation of the results shown in Figs. 1 to 3 could be considerably improved. For one thing, the growth differences between the various strains are not evident in Fig. 1C, Fig. 2B and E, and
Fig. 3B, although the triple mepΔ mutants should not grow on SLAD medium. I assume that the triple mepΔ mutants and some of their derivatives formed tiny colonies, and the pictures were taken at
different magnifications to show the colony borders, although this is nowhere stated in the manuscript. Growth differences are illustrated in Fig. 1A, Fig. 2A and D, and Fig. 3A, but these pictures appear not
very reliable, as is evident from the fact that the mep2Δ single mutant seems to have a growth defect on SLAD medium that is rescued after reintroduction of wild-type MEP2 (a growth defect of the mep2Δ single mutant is unexpected, because previous studies reported that Mep1 alone is sufficient to support wild-type growth). I suggest the following modifications to these experiments and their presentation:

a) Growth and filamentation of the various strains on solid media should be simultaneously illustrated by showing representative single colonies of all strains at equal magnification. I notice that the filamentation phenotype of the wild type on SLAD medium was generally weak in the
authors' experiments, with only occasional pseudohyphae at the colony borders, whereas in other studies, S. cerevisiae colonies grown on SLAD plates are completely surrounded by a filamentous fringe (see, for example, the original study by Lorenz and Heitman describing the role of
Mep2 in filamentous growth).

There is a misunderstanding on the way we do the filamentation tests. We are sorry for that and understand that this can have raised doubts regarding our conclusions. As described in the materials and methods section, the filamentation and the growth tests are not done in the same way. The filamentation pictures do not correspond to a different magnification of one colony selected amongst those arising from isolated cells streaked on SLAD plate photographed for the growth test. For filamentation tests, we drop a high-density” cell suspension in a small area in the plate (drop tests). For the growth tests, to estimate the capacity of cells to grow, we streak a low-cell-density suspension on a plate, in a way to obtain isolated clones. This has been detailed in the Material and Methods. We never observed growth of the triple-*mep∆* cells on SLAD, as shown in Fig.2A (former Fig.1A). Former Fig1C (new Fig.2B), Fig.2B (new Fig.3B), Fig.2E (new Fig.3E) and Fig.3B (new Fig.4A) represent filamentation “drop” tests. In these experiments, the triple-*mep∆* cells do not grow, but the dropped cells are visible. This method enables to have a standardized procedure to follow filamentation in a more homogenous way. We conclude by comparing the filamentation capacities of each variant in the same unique experiment by comparing with native Mep2. Following the filamentation by surveying the growth tests is more difficult to interpret as different degrees of filament production occur on colonies selected in dense regions or borders of the streaked zone (inhomogeneity considering the different isolated colonies).

We are grateful to the Reviewer for the comment concerning *mep2∆* cells that did not grow on SLAD (former Fig 2A). This is due to a mistake during the experiment or mounting of the picture and we unfortunately did not see the obvious bug in the submitted manuscript. We repeated the growth test shown in former Fig. 2A (new Fig. 3A). In this new experiment, all the controls behave as expected. We are sorry for this mistake and understand that this can have raised doubts regarding our conclusions.

b) The effect on filamentation by all Mep2 variants should also be tested in a mep2Δ single mutant background (as has been done for some mutants in Fig. 2B) to exclude an influence of reduced growth on filamentation.

We agree. The incapacity of different Mep2 variants to induce filamentation has now been also tested in *mep2∆* cells (S1 Fig). The behaviors are the same as those observed in the triple-*mep∆* background. This is now also mentioned in the text (lines 162-164, 246-247, 337-338, 348-350).

c) The doubling times of the strains in liquid SLAD medium should be determined to allow a better quantification of the effect of the different Mep2 mutations on growth for comparison with the effect on ammonium transport, which was also determined for cells growing exponentially in liquid medium.

We understand the point raised by the Reviewer. Unfortunately, we are again blocked by our inability to reproduce in liquid medium the filamentation conditions reached on solid SLAD after several days of growth. SLAD contains the initial concentration of 100µM ammonium, a limiting concentration that is rapidly consumed during growth. To measure the doubling time in liquid medium, we would need to reach the balanced exponential phase of growth. This is not possible with 100µM ammonium that would be rapidly consumed during growth.

We performed the ammonium removal assays in SHPD, where proline is provided in a non-limiting concentration to be able to reach the balanced exponential phase of growth and work in reproducible conditions.

d) Finally, I found it very difficult to find the data for some strains in Fig. 1B, because the symbols cannot be easily distinguished. This could be amended by using colored symbols.

As suggested by several Reviewers, we split and added colors to the graphs in former Fig. 1B, new Fig 1C.

3) I think that the authors should tone down their conclusion in the abstract that the C-terminal tail of Mep2 is not required for the induction of filamentation. Despite the fact that the additional H199Y
mutation in the truncated Mep2S426stop protein restored ammonium uptake to wild-type levels (Fig. 3C), filamentation was extremely poor (Fig. 3B). Therefore, the function of the C-terminal tail is not only to upregulate substrate translocation, but it is also required for normal signaling at wild-type transport levels. Signaling may require interactions between the C-terminal tail and other cytoplasmic domains of Mep2 (this has been suggested for C. albicans Mep2), and conformational changes caused by the H199Y mutation may have allowed some signaling to occur in the absence of these interactions (or allowed such interactions to occur with the rest of the C-terminal tail that remains in the truncated protein).

We agree with the Reviewer. We reviewed the text to be more cautious with this conclusion (lines 46-48, 241, 264-266, 533-539).

4) The H194E mutation is highly interesting in that it abolishes filamentation, although it increases ammonium transport, a striking exception to the generally observed correlation between ammonium
transport and signaling capacity by Mep2. The authors argue that the H194E mutation altered the mechanism of ammonium transport by Mep2 into a Mep1-like mechanism, as already suggested in their previous study. Unfortunately, this mutated protein could not be successfully produced
in oocytes, which would have allowed to test if the mutation changed ammonium transport from electroneutral to electrogenic (as a minor note, the sentence in lines 397-399 should be changed, because the H194E variant was not tested in the experiments shown in Fig. 6E).

The sentence was changed.

As this is a critical aspect of the present study, I think that the authors should at least test if Mep2 with the H194E mutation shows a Mep1-like behavior instead of a Mep2-like behavior with regard to cytosolic
alkalinization (see Fig. 5). If this test can reliably distinguish between the two transport mechanisms, the authors could prove or refute this hypothesis.

We agree. As detailed in the response to point 1 of Reviewer 1, we designed an experiment to assess the submembrane, local, consequence of substrate transport via Mep proteins by directly fusing pHluorin at their C-terminal extremity. The fluorescence emission by Mep1-pHluorin was too low to serve as a reporter of translocation activity, most likely due to the low expression level of the chimera reached with the natural *MEP1* promoter. By contrast, Mep2-pHluorin expressed under the control of the *MEP2* promoter was localized at plasma membrane and produced a specific emission signal. In cells expressing the inactive Mep2^D186N^-pHluorin, addition of ammonium (2 or 0.5 mM) had no clear impact on the submembrane pH, consistent with the inability of Mep2^D186N^ to recognize and transport ammonium (Fig. 7B). In cells expressing Mep2-pHluorin, ammonium addition, either at 2 or 0.5 mM, induced an initial acidification phase followed by a rapid alkalinisation phase (Fig.7A). Of note, in cells expressing Mep2^H194E^-pHluorin, addition of 2 or 0.5 mM ammonium induced a pronounced transient drop of pH followed by a pH recovery phase (Fig.7C). The drop reached 0.1 unit and was even bigger than the one observed for cytosolic pH in cells expressing Mep1 (Fig.6C). These results suggest that Mep2 and Mep2^H194E^ could transport ammonium through different mechanisms.

5) Although the study focuses on Mep2 of S. cerevisiae, the paper implies that the conclusions about Mep2 function derived from this study extend to its homologs in other fungi. Nevertheless, the authors point out striking differences between the Mep2 proteins of S. cerevisiae and C. albicans, which may hint to different signaling mechanisms by the two proteins. In C. albicans, the C-terminal cytoplasmic tail of Mep2 is required for the induction of filamentous growth even when ammonium transport by mutant proteins appears normal. Mep2 proteins that were C-terminally truncated after amino acid 440 and 423 allowed wild-type growth and exhibited comparable ammonium transport activity.
However, while the former promoted hyperfilamentation (caused by the absence of an autoregulatory domain, increased expression levels, or both), the latter was unable to induce filamentation. According to the model proposed in the present study, the removal of the 17 additional amino acids should have altered the mechanism of ammonium transport (to a Mep1-like mechanism) to explain the loss of signaling. The C. albicans mutants are available and could be tested and compared in the same way as the S. cerevisiae mutants. Since the authors repeatedly highlight the relevance of the transition from yeast to filamentous growth for the virulence of pathogenic fungi (and our understanding of its regulation), it seems worthwile to perform and include these experiments.

We agree with the Reviewer that it would be informative and nice to make a comparative analysis on the role of ScMep2 and CaMep2. Our lab has always focused on the characterization of the *S. cerevisiae* behavior, while other experts in the world focused on other specific fungi. We would prefer not to interfere with their lines of research.

We nevertheless designed a few experiments to address the behavior of CaMep2 expressed in *S. cerevisiae*. The team of Pr. Morschhäuser has previously proposed that the signaling via CaMep2 in *Candida* *albicans* might be controlled in different ways compared to signaling via ScMep2 in *S. cerevisiae* (Neuhäuser et al., 2011). In Biswas and Morschhäuser (2005), this team showed that CaMep2 is able to allow filamentation in *S. cerevisiae* while CaMep1 is not, consistent with the idea that CaMep2 and ScMep2 signaling could proceed in similar ways. Here, we constructed vectors to express and overexpress CaMep2 in *S. cerevisiae* under the control of the *MEP2* promoter from *S. cerevisiae*, and addressed the impact of the H188E pore mutation corresponding to H194E in ScMep2. We in parallel constructed vectors with these proteins directly fused to pHluorin with the hope to measure the impact of their activity on submembrane pH.

We show that CaMep2 and CaMep2^H188E^ expressed from a centromeric plasmid do not efficiently complement the growth defect of triple-*mep∆* cells, growth being only barely detected after 7 days on SHAD (Fig 9A). No filamentation was observed in these conditions (Fig 9B). We were not able to observe by microscopy plasma membrane fluorescence using the pHluorin fusions in proline medium.

Expressing the *C. albicans* Mep2 variants from an episomic plasmid allowed a better complementation of triple-*mep∆* cells, growth being visible after 7 days on both SLAD and SHAD (Fig 9A). On SLAD, filaments were observed around the drop of cells overexpressing CaMep2 but not with CaMep2^H188E^. On SHAD, filamentation was also observed around the cells overexpressing CaMep2 (Fig 9B). This is consistent with our unpublished data and data from Rutherford and colleagues showing that ScMep2 overexpression allows filamentation even on SHAD (Rutherford et al., 2008). Of note, CaMep2^H188E^ overexpression allowed efficient growth but no filamentous growth indicating that the H188E mutation impairs signaling (Fig 9). These results indicate that the mutation of the histidine into glutamate has a similar impact on both ScMep2 and CaMep2, both proteins conserve transport activity but loose signaling capacity. Unfortunately, even if some fluorescence was detectable at the surface of a fraction of cells using the CaMep2 pHluorin fusions expressed from the episomic plasmids, this was inhomogeneous and the emitted fluorescence was unfortunately too low to properly record pH variations as illustrated in the figure below.

We have included the CaMep2 experiments in the revised manuscript (Figure 9).

We did not try to record CaMep2^H188E^ activity in Xenopus oocytes as we know that ScMep2^H194E^ is not stable in oocytes, and this appears to be also true for several plant AMTs with similar substitutions in the pore-facing histidines (Neuhäuser personal communication, Ganz et al., in revision). Another indication of a similar behavior of ScMep2 and CaMep2 is given by electrophysiological experiments in *Xenopus leavis* oocytes, characterizing CaMep transport mechanisms. Similar to the situation in *S. cerevisiae*, CaMep1 mediates electrogenic substrate transport while CaMep2 shows electroneutral net ammonia uptake (Neuhäuser, unpublished).

We can’t exclude that dedicated regions of the Mep2 C-terminal tail are involved in a switch of transport mechanism. What we may say is that the ScMep2 ^C∆434-485^ variant, that lacks the sequence corresponding to the region of the CaMep2 C-terminus that appears required for filamentation, is still able to allow efficient filamentation in Npr1-lacking cells (Fig. 2B).

Reviewer #4: In this manuscript the authors build on their previous studies of the Mep2 transceptor from Saccharomyces cerevisiae. Three main findings are presented: that signalling is linked to transport activity, that the Mep2 CTD is not required for signalling and that signalling involves pH changes within the cytosol. In support of the latter, the authors present evidence that the Mep2 transceptor and the non-signalling Mep1 transporter induce different cytosolic pH profiles. Furthermore, the authors establish that transport by Mep2 is electroneutral whereas transport by Mep1 is electrogenic. Together these data support the model that Mep2 imports ammonia gas that raises cytosolic pH. This is an important finding. What is disappointing is that the authors have not gone one step further and tested some predictions of this model. For example, if the Mep2 dependent pH change regulates signalling then Mep2 variants that transport but do not signal should generate a different pH profile. This may be because transport by the variant is electrogenic or if it remains electroneutral the amount of imported ammonia differs from that imported by wild-type Mep2. Too
little or too much ammonia may result in a pH change that does not support signalling. The authors could test the effect on cytosolic pH of the Mep2H194E variant as they have determined that transport by it is electroneutral. Similarly, the hyperactive Mep2G349C variant would be expected to generate a different pH profile from that of the Mep2H194E variant. Other points of discussion could include those Mep2 homologues that act as signalling proteins in S. cerevisiae, such as Ump2 from U. maydis. These should have the same impact as Mep2 on cytosolic pH via electroneutral transport when expressed in S. cerevisiae.

As detailed in the response to point 1 of Reviewer 1, we addressed the point raised by this Reviewer by constructing plasmids allowing to express Mep proteins directly fused to the pHluorin. As shown in Fig.7, ammonium transport associated to Mep2^H194E^-pHluorin induces an important cytosolic acidification compared to what is observed with Mep2-pHluorin. These results suggest that ammonium transport via Mep2 and Mep2^H194E^ has a different impact on pH_c_ in the tested conditions.

To address the impact on pH_c_ of the transport activity of Mep2 homologues expressed in *S. cerevisiae*, we constructed vectors enabling to express under the control of the baker yeast *MEP2* promoter several variants of *Candida albicans* Mep2 fused to pHluorin. Unfortunately, the fusion proteins did not produce a sufficient specific fluorescence emission to perform kinetic experiments.

Nevertheless, we show that CaMep2^H188E^ overexpression allowed efficient growth but no filamentous growth indicating that the H188E mutation impairs signaling (Fig 9). These results indicate that the mutation of the histidine into glutamate has a similar impact on both ScMep2 and CaMep2, both proteins conserve transport activity but loose signaling capacity.

In addition, electrophysiological experiments in *Xenopus leavis* oocytes, characterizing CaMep transport mechanisms show that similar to the situation in *S. cerevisiae*, CaMep1 mediates electrogenic substrate transport while CaMep2 shows electroneutral net ammonia uptake (Neuhäuser, unpublished).

Minor comments

lines 131-198

The fact that certain Mep2 variants are more active in the absence of Npr1 may be due to their increased expression and could be tested. A previous study by the authors (Boeckstaens et al., 2013) established that the Mep2 levels are higher in the absence of Npr1.

Indeed, it is known that, in the absence of Npr1, genes submitted to nitrogen catabolite repression, such as *MEP2*, are upregulated (Feller et al., 2006). The Mep2 signal appears more intense in the absence of Npr1 (Fig 2C, Boeckstaens et al., 2014). These aspects have been commented in the revised manuscript (lines 497-506).

lines 191-131

The authors conclude that the Mep2S457D variant does not lock Mep2 into a constitutively active conformation as it does not induce filamentation when cells are grown on SHAD plates. Expression of this variant would be expected to be reduced under SHAD conditions. Wild-type Mep2 needs to be
highly expressed if it is to signal in response to low ammonium. A variant locked in a constitutively active conformation may therefore loose its ability to signal if expressed at a low level. The relative
levels of Mep2S457D in response to SLAD and SHAD should be determined.

We understand the general point raised by the Reviewer. However, we would like to precise that what we conclude regards signaling: “**The S457D phosphomimetic mutation does not lock Mep2 in a conformation constitutive for signalling**”. Our conclusion is built on several observations. Mep2S457D allows filamentation like Mep2 on SLAD and we see no hyperfilamentation in this condition that could reflect an increased/sustained signaling activity. Consistent with this, there is no filamentation on SHAD. Note that a H199Y variant is able to produce filamentation tips even in SHAD, indicating that repression is not totally precluding filamentation on SHAD. Moreover, even if we adopted a fixed protocol in which we observe filamentation at day 7 for overall comparisons, we use to follow the plates beyond this time point. At late stages, Mep2 is even able to allow filamentation on SHAD. And even in these conditions, no particular phenotype of S457D variant was observed. Mep2^D186N,S457D^ does not allow filamentation on SHAD, not even on SLAD, even in cells in which expression of Mep1 and Mep3 is ensuring growth. So in the latter conditions, if the S457D mutation would lock Mep2 in a constitutive signaling conformation, we would expect to see filamentation.

*MEP2* gene responds to the nitrogen catabolite repression, its expression being reduced in the presence of a preferential nitrogen source present in a sufficient quantity. SHAD condition, containing 1 mM ammonium, is considered as a derepression condition. It is difficult to measure expression levels in liquid SHAD or SLAD conditions because we cannot reach a balanced phase of exponential growth, the nitrogen source will be rapidly exhausted during growth so that at the end what we will measure is expression in nitrogen starvation conditions. In Marini et al., 1997, we performed a switch from proline medium to low ammonium containing media to address the *MEP2* level of expression in a transient way during ammonium limitation. Clearly, ammonium 1mM or 0.5mM behaved as proline, thus as nitrogen derepressing conditions. We here nevertheless checked that the S457D variant is not generally more sensitive than Mep2 to NCR due to potential better uptake of ammonium. As shown here after, Mep2^S457D^ is not less produced than Mep2 even in the presence of 20 mM ammonium.
