# Supplementary material for: Yeast filamentation signaling is connected to a specific substrate translocation mechanism of the Mep2 transceptor
Source: PLoS Genet. 2020 Feb 18;16(2):e1008634. doi: 10.1371/journal.pgen.1008634 (PMC7048316; doi:10.1371/journal.pgen.1008634)
Supplement: Attachment — Submitted filename: Revision letter.docx [file pgen.1008634.s008.docx]

Reviewer #1: I am impressed by the extensive and thoughtful changes to the manuscript in response to the previous suggestions, particularly the clever pHluorin fusion experiments. I have no further reservations regarding the manuscript, though the authors may want to re-edit the paragraph at the bottom of page 17, as there are several typos.

We thank the Reviewer, we corrected the typos that we detected.

Reviewer #2: The authors nicely addressed all of my comments in this revised manuscript. I do not have any more comments. Nice work!

We thank the Reviewer.

Reviewer #3: In their revised manuscript, the authors went to great lengths to address the reviewers' comments. Therefore, I would not request additional experiments, but there are several issues that in my opinion should be clarified.

We thank the Reviewer for the deep and careful analysis of the data and for not requesting more experiments at this stage. We hope to be able to fully address specific issues in future work.

1) I still have difficulties to see how the short-term changes in intracellular pH that were observed upon addition of ammonium to proline-grown cells can be responsible for the induction of filamentous growth after several days under nitrogen-limiting conditions, as the cells should re-establish and maintain intracellular pH homeostasis.

We didn’t write that the changes in intracellular pH observed upon addition of ammonium to proline-grown cells are responsible for the induction of filamentous growth. The data obtained with cells growing exponentially in liquid buffered non-limiting proline medium after addition of ammonium 0.5 mM can’t be directly compared to the filamentation growth observed after several days on solid non-buffered limiting SLAD medium with initial concentration of ammonium of 0.1 mM. We now underline this point (lines 151-153). How pH homeostasis is controlled in the latter conditions over time is not known. Substrate translocation via Mep1 and Mep2 differently impacts on intracellular pH, at least in the tested *in vivo* conditions. The Mep2^H194E^ pore variant shows net alteration in the way substrate translocation impacts on intracellular pH, and this variant is unable to allow filamentation.

Moreover, electrophysiological data in the heterologous oocyte expression system are consistent with a different substrate transport mechanism between Mep2 (electroneutral) and Mep1 (electrogenic). We observed correlations (connections as indicated in the title) between the used substrate translocation mechanism and the capacity to induce filamentation. Solving the precise signalling mechanism by which Mep2 allows filamentation will require further investigation.

As mentioned before, the ideal experiment would be to measure Mep2 activity and pH modifications in conditions of filamentation, so in cells growing for several days on SLAD, thus largely starved for nitrogen. This is technically very difficult to design. The MEP2 gene, which responds to the nitrogen catabolite repression, is probably more expressed in condition of filamentation than on the proline medium used to measure pH changes. This overproduction of the protein may lead to an accentuated transport of NH_3_, leading to a more important alkalinisation. If we follow the SHAD plates for a longer period than 7 days, filamentation also occurs in a Mep2-dependent manner. Also, if Mep2 is overexpressed, or expressed under the GAL1 promoter on SHAD, this gives rise to clear filamentation (Rutherford et al., 2008, and our unpublished data). If we consider that Mep2 leaves the proton in the periplasm as part of the transport mechanism, this could also constitute an advantage for cells quasi-starved for nitrogen. This could contribute to maintain an electrochemical gradient in turn favoring the transport of other surrounding molecules.

The authors may also explain why there is an initial drop in intracellular pH upon ammonium uptake, the reason for which is not clear to me.

The reason of the initial drop upon ammonium addition is not clear. This instant and transient acidification is observed with native Mep2 directly fused to the pHluorin and not in yeast expressing cytosolic pHluorin for instance. This very fast change in fluorescence ratio could be linked to a conformational change in Mep2 induced by ammonium addition and in turn impacting on the pHluorin moiety. Additional experiments would be necessary to address this question. We could fuse pHluorin to another abundant plasma membrane protein via chromosomal insertion and monitor the impact of ammonium addition in cells expressing native Mep2. We could also use this system to address the impact of ammonium transport via other Meps or variants on intracellular pH.

Although Mep1 transports the proton together with ammonia, one would expect that ammonia is immediately protonated again within the cell, so that there is no net increase in the intracellular concentration of protons by Mep1-mediated ammonium transport.

In the case of Mep2, ammonia transport without the dissociated proton should cause an initial intracellular alkalinisation, because cytosolic protons are captured by the internalized ammonia. Which mechanisms are responsible for the actually observed pH changes?

In the case of Mep1, NH_3_ will in principle be rapidly protonated after transport to form NH_4_^+^ and a part of NH_4_^+^ will dissociate into H^+^ and NH_3_, leading to cytosolic acidification (like the acidification that would be transiently observed if NH_4_^+^ was transported). Transport through Mep1 and Mep2^H194E^ are rather characterized by a prolonged cytosolic acidification and a recovery phase.

In the case of Mep2-phluorin, as mentioned before, the initial and transient instant acidification observed could be linked to a conformational change affecting the pHluorin directly fused to Mep2 and in turn altering the fluorescence ratio. Besides this rapid, transient acidification, alkalinisation (trespassing of the initial pH value) is observed with Mep2-pHluorin.

The interpretation of the *in vivo* pHluorin experiments is delicate as the curves somehow reflect multiple parameters englobing substrate(s) transport, dissociation, diffusion processes, trapping processes, buffering, transport inactivation processes, compensation via Pma1, and also ammonium metabolism by Gdh and GS.

We were very cautious with our conclusions in the manuscript and mainly described that Mep1 and Mep2-mediated translocation of substrate(s) have specific different impacts on pHc, and that transport activity of Mep2^H194E^ appears to impact intracellular pH in a similar way than Mep1 activity. These data, proposing a different transport mechanism for Mep1 and Mep2, were further sustained by the electrophysiological data.

2) The authors point out that some Mep2 CTD variants showed an even better complementation efficiency in the absence of Npr1 than in its presence, and that this correlated with an improved ammonium removal rate in SHPD and also with an increased capacity to induce filamentation on SLAD (Page 9). This appears to be the case for the variants shown in the upper panels of Fig. 1C. However, the Δ442-449, Δ442-485, and Δ450-485 variants, which all confer a strong hyperfilamentous phenotype in the absence of Npr1 (Fig. 2B) do not show better ammonium uptake in the absence of Npr1 than in its presence (Fig. 1C, lower panels). If anything, the uptake rate is slightly lower in the absence of Npr1 (this is now better visible in the revised figure). Therefore, stronger filamentation does not correlate with more efficient ammonium uptake in these cases.

We agree that the correlation is not visible for all variants. This is why we wrote ‘Several variants…’ in the text. We now better specified the concerned variants in the manuscript (lines 187-188, 202-209).

We agree that for the Δ442-449, Δ442-485, and Δ450-485 variants, the hyperfilamentous phenotype in Npr1 absence appears not correlated to an improved ammonium removal in SHPD compared to Npr1-containing cells. Npr1 absence *per se* might have a positive impact on filamentation efficiency. Note that considering only *npr1* cells, the Δ442-449, Δ442-485, and Δ450-485 variants conferred some growth advantage compared to the other variants in the positive domain, visible in Fig. 2A SHAD day 3 in *npr1* cells. Also, as referred for the Δ450-485 variant in the manuscript (line 183), in Boeckstaens et al., 2014, initial rates of ^14^C-methylammonium uptake showed those three variants to be more active in Npr1-lacking cells.

3) In a similar vein, the repeatedly emphasized correlation between transport and filamentation efficiencies does also not hold in several other cases. The H199Y S426stop mutation did not impair transport (it was even slightly increased, Fig. 4C), but almost completely abolished filamentation (Fig. 4A, top panels). Furthermore, the G349C mutation strongly increased transport (Fig. 4D) without resulting in enhanced filamentation in NPR1 wild-type cells (Fig. 4A, top panels). This should be more critically discussed.

We agree with the point that Mep2^H199Y,S426stop^ appears not affected in ammonium removal in SHPD but induces an altered filamentation. For the second example, we published in Boeckstaens et al., 2007 that the Mep2^N4Q,G349C^ variant shows increased Vmax and rather similar Km in *npr1* cells compared to Npr1^+^ cells. The Vmax was even better than the one of Mep2^N4Q^ (N4Q used to avoid glycosylation smear on western blot), but Km was slightly affected. Mep2^N4Q,G349C^ allowed hyperfilamentation in *npr1* cells but also increased filamentation in Npr1^+^ cells. Here, we used the simple G349C variant that indeed shows increased ammonium (0.5 mM) removal in SHPD compared to native Mep2. There is a growth advantage with this variant visible on ammonium 1mM (Fig. 4A SHAD, day 7). But this advantage is not visible on SLAD (ammonium 0.1mM), probably due to a lowered affinity for ammonium, and we observed no enhanced filamentation.

We notably mentioned in the former manuscript that we do not exclude that the C-terminal extremity of Mep2 somehow plays a positive role in the filamentation process.

We already acknowledged the existence of several situations concerning the correlation between transport and signaling efficiencies in the introduction section (lines 97-101). We tried to still better clarify these points in the manuscript (lines 279-280, 303-308).

4) In my previous comments I had suggested that the authors compare the mode of ammonium transport by two different C-terminally truncated C. albicans Mep2 proteins, one of which promotes strong hyperfilamentation, while the removal of few additional amino acids abolished filamentation without altering transport efficiency. According to the model proposed in the present study, the additional truncation should have altered the mode of transport from Mep2-like to Mep1-like.

We agree and added the hypothesis in the discussion section (lines 630-634). Having set up the ScMep2-pHluorin assay and knowing the H194E mutation impact on pHc prompted us to construct CaMep2-pHluorin to effectively be able to address the impact of C-terminally truncated variants on pHc. Unfortunately, as mentioned in the previous response to the Reviewers, we could not use the CaMep2-pHluorin fusions to record pH as the fluorescence was inhomogeneous and too low.

We hope to address this point in future work.

Instead, the authors compared wild-type CaMep2 and a mutated form, in which H1 of the conserved histidine-twin in the conducting pore was changed into glutamate found in Mep1-like proteins, upon heterologous expression in S. cerevisiae. The finding that the mutation abolished filamentation is taken as evidence that a conserved transport mechanism explains the ability of both ScMep2 ans CaMep2 to promote filamentous growth. However, the authors disregard (see line 451) that the mutated Mep2 restored growth of SCmepΔ triple mutants on SLAD plates much less efficiently than did wild-type CaMep2 (Fig. 9A). It is likely that the mutated protein was less stable than the wild-type protein, explaining its inability to promote filamentation.

As we were not able to record CaMep2-pHluorin, we decided to test the impact of H188E mutation on CaMep2 ability to allow filamentation in *S. cerevisiae* triple-*mepΔ* mutant to nevertheless address the impact of the translocation mechanism on filamentation induction by CaMep2. We agree that the CaMep2^H188E^ restored growth of *S. cerevisiae* triple-*mepΔ* mutant on SLAD plates less efficiently than did wild-type CaMep2. However, on SHAD plates, the growth ensured by CaMep2^H188E^ is equivalent to the one ensured by native CaMep2, and cells expressing the CaMep2^H188E^ variant were not able to induce filamentation contrary to the one expressing native CaMep2. We specified in the text (line 474) that it was observed in the SHAD conditions.

Reviewer #4: Associate editor note, this reviewer did not have detailed comments for the authors and found the manuscript improved and suitable for acceptance.
We thank the Reviewer.
